# Functional Polymer Catalysts for CO_2_ Capture and Conversion: Design Principles, Applications, and Future Perspectives

**DOI:** 10.3390/polym18192337

**Published:** 2026-09-25

**Authors:** Saikumar Manchala, Deshetti Jampaiah, Paramita Koley, Aristides Bakandritsos, Suresh K. Bhargava

**Affiliations:** 1 Regional Centre of Advanced Technologies and Materials, Czech Advanced Technology and Research Institute, Palacky University, Olomouc, Slechtitelu 27, 779 00 Olomouc, Czech Republic; saikumar.researcher@gmail.com (S.M.);; 2 Centre for Advanced Materials and Industrial Chemistry (CAMIC), RMIT University, Melbourne, VIC 3000, Australia; 3 Nanotechnology Centre, Centre for Energy and Environmental Technologies, VSB-Technical University of Ostrava, 17 Listopadu 2172/15, 708 00 Ostrava, Czech Republic

**Keywords:** functional polymer catalysts, CO_2_ capture, CO_2_ conversion, cyclic carbonates, CO_2_ hydrogenation, porous organic polymers, carbon capture and utilization, green chemistry

## Abstract

Functional polymer catalysts have emerged as a transformative class of materials for CO_2_ capture and conversion, offering exceptional tunability, high CO_2_ affinity, and excellent recyclability. This review provides a comprehensive polymer-centric analysis of four major classes of functional polymer catalysts—linear and soluble polymers, crosslinked networks and resins, porous organic polymers (POPs), and polymer–metal hybrids—with a unified focus on their roles in CO_2_ capture, CO_2_ cycloaddition to epoxides, CO_2_ hydrogenation, and integrated carbon capture and utilization (CCU) strategies. We discuss how the polymer backbone chemistry, active site density, microenvironmental effects, hydrophilic–hydrophobic balance, and pore confinement collectively govern the CO_2_ uptake capacity, activation, conversion, and selectivity of these materials. The stability, recyclability, and green metrics of the polymer catalysts were critically assessed, and their performance was benchmarked against that of inorganic supports, metal–organic frameworks (MOFs), and covalent-organic frameworks (COFs). Current challenges, including structure–activity gaps, operando characterization, standardized performance reporting, processability, and scale-up, are also discussed.

## 1. Introduction

The accelerating accumulation of atmospheric CO_2_, driven by fossil fuel combustion and industrial processes, is one of the most pressing challenges of our time. Mitigating CO_2_ emissions while simultaneously extracting value from captured CO_2_ requires efficient materials that can selectively concentrate CO_2_ from dilute streams and, in integrated systems, catalytically convert the captured carbon into value-added products, such as cyclic carbonates (CCs), formate, methanol, CO, and several useful chemicals and fuels [1,2]. In this context, functional polymer catalysts have emerged as a uniquely versatile platform that bridges CO_2_ capture and catalytic conversion within a single, recyclable material framework [3,4].

Traditional CO_2_ capture approaches based on aqueous amine scrubbing suffer from high regeneration energy costs, corrosion, and amine degradation. Solid sorbents based on inorganic supports (zeolites, silica, and activated carbon) offer improved stability but limited tunability of amine loading and pore architecture [5]. Functional polymer catalysts can overcome many of these limitations. Functional polymer catalysts are a new variety of materials composed of catalytically active functional groups integrated within polymer frameworks or dispersed metal centers, or sometimes both, which engage immediately in chemical reactions owing to their versatile properties, including structural and thermal stability, chemically tunable functionalities, high porosity, efficient catalyst recovery, and metal-anchoring capability. The density, type, and spatial arrangement of CO_2_-binding functional groups (amines, ionic liquids, triazines, and Lewis acid sites) can be precisely tuned by monomer selection, polymerization method, and post-synthetic modification. Moreover, appropriately designed polymer frameworks with multifunctional properties and supported metal centers can host both CO_2_-capturing groups and catalytic sites, enabling integrated CCU and conversion without a separate CO_2_ desorption, purification, or compression step [6,7,8].

Functional polymer catalysts, mainly POPs, including conjugated microporous polymers (CMPs), hypercrosslinked polymers (HCPs), and covalent triazine frameworks (CTFs), have attracted particular attention owing to their permanent microporosity (BET surface areas of 500–3000 m^2^ g^−1^), tunable pore chemistry, and outstanding chemical stability under CO_2_ conversion with appropriate linkages [9,10]. Polymer–metal hybrid systems extend the accessible CO_2_-conversion chemistry by supporting transformations, including hydrogenation and electrochemical or photochemical reduction, which require metal active sites [11,12].

Despite the rapid growth of this field, existing reviews either focus narrowly on CO_2_ adsorption by porous polymers [13,14] or on POPs for the electro-and photocatalytic reduction of CO_2_ [15] or on POPs for catalytic cycloaddition chemistry without providing a unified polymer-design-centric perspective that connects the material structure to a broad range of CO_2_ capture and conversion applications [15,16,17]. Therefore, the studies discussed were selected primarily based on their connection to the thematic focus of this article, with special attention to works dealing with key developments in the design, synthesis, catalytic activity, and mechanistic understanding of functional polymer-based catalysts for CO_2_ capture and conversion. Preference was given to recent influential reports and highly cited representative publications that emphasized basic insights or demonstrated seminal improvements in catalytic efficiency, selectivity, stability, and structure-property relationships. Furthermore, we present a comprehensive analysis of functional polymer catalysts, with an emphasis on the CO_2_-relevant functional groups and polymer-design principles that govern the capture, activation, mass transport, selectivity, and catalyst lifetime across the major reaction classes considered. The stability, recyclability, sustainability, current challenges, and research priorities of catalysts are also evaluated.

## 2. Classes of Functional Polymer Catalysts

The principal classes of functional polymer catalysts applied for CO_2_ capture and conversion are summarized in Table 1 and Scheme 1, respectively. Four major classes are distinguished by their architecture, porosity, and nature of their CO_2_-active functional groups [18,19,20].

### 2.1. Linear and Soluble Functional Polymers

Linear and soluble polymers are long and unbranched polymeric materials with covalently bonded highly reactive functional groups or CO_2_-capturing groups with solution dispersibility, including primary and secondary amines, guanidinium units, and carbonate precursor moieties, which can be applied as homogeneous-phase CO_2_ sorbents in liquid-phase scrubbing systems. Poly(ethylenimine) (PEI) and poly(allylamine) in solution achieve rapid CO_2_ absorption kinetics owing to their high local amine density and the absence of diffusion barriers [18,21]. A prominent advantage is the near-homogeneous microenvironment around each active site; however, the post-absorption separation of the polymer sorbent from the CO_2_ gas stream and the recovery of the polymer and release of the bound CO_2_ require additional separation and regeneration steps compared to those of fixed solid sorbents [18].

### 2.2. Crosslinked Polymer Networks and Resins

Crosslinked polymer networks are three-dimensional and covalently or dynamically interconnected polymeric materials, most of which are based on divinylbenzene (DVB)-crosslinked polystyrene or polyacrylate matrices. They offer mechanical robustness and compatibility with fixed-bed flow reactors, making them well-suited for continuous CO_2_ capture and conversion. Amine-functionalized crosslinked resins, prepared by post-sulfonation and amination or by direct copolymerization of amine-bearing monomers with DVB, achieve CO_2_ uptake capacities of 1.5–2.5 mmol g^−1^ at 0.15 bar and can be thermally regenerated at 90–110 °C [22,23]. Imidazolium-functionalized crosslinked polymers serve as heterogeneous ionic liquid analogs for CO_2_ cycloaddition to epoxides, providing halide nucleophiles required for epoxide ring opening in a recoverable solid form [24]. The degree of crosslinking critically affects the swelling, internal pore accessibility, and proximity of cooperating active sites [25].

### 2.3. Porous Organic Polymers

POPs are low-density, permanently porous, and three-dimensional polymeric materials, including HCPs, CMPs, CTFs, and polymers of intrinsic microporosity (PIMs), and constitute the most intensively researched class of materials for CO_2_ applications owing to their ultra-high BET surface areas (500–3000 m^2^ g^−1^) and tunable microporosity [9,10,26]. HCPs are generally prepared by Friedel–Crafts hypercrosslinking and they are particularly attractive as CO_2_ sorbent supports. For example, amine-grafted HCPs achieve CO_2_ uptake capacities of 3–4 mmol g^−1^ at 0.15 bar, which is among the highest values reported for solid amine sorbents [27]. CTFs, prepared by the trimerization of aromatic nitriles under ionothermal conditions, offer intrinsic Lewis basic triazine N sites with high isosteric heats of CO_2_ adsorption (25–35 kJ mol^−1^) and remarkable hydrothermal stabilities [28]. CMPs, assembled via the transition-metal-catalyzed coupling of multifunctional monomers, allow the co-incorporation of photosensitizer units and CO_2_ reduction catalysts within the same porous network, enabling photocatalytic CO_2_ conversion [29,30].

### 2.4. Polymer–Metal Hybrid Catalysts

Polymer–metal hybrid catalysts are important polymeric materials essential for CO_2_ transformations. They require anchored transition metal active sites for CO_2_ hydrogenation to formate or methanol, CO_2_ electroreduction, and photocatalytic CO_2_-to-CO conversion. To design them, three principal strategies are employed including (i) immobilization of discrete metal complexes via chelating pendant ligands (pyridyl, phosphino, NHC, bipyridyl, and pincer ligands) grafted to the polymer backbone [8,31], (ii) encapsulation of metal nanoparticles (Cu, Ru, Ni, and Fe) within POP matrices to prevent aggregation under reaction conditions [32], and (iii) in situ formation of metal clusters within ionic polymer frameworks, exploiting ion exchange to introduce the metal, followed by reductive activation [33]. The polymer matrix modulates the electronic density at the metal center through ligand field effects and neighboring functional group interactions, providing an additional handle for tuning the CO_2_ activation selectivity. For example, this type of modulation can steer towards formate vs. methanol vs. CO in CO_2_ hydrogenation reactions [34].

**Table 1:** Classification of functional polymer catalysts: representative functional groups, synthesis routes, advantages, and limitations.

Class	Representative Functional Groups	Common Synthesis Routes	Key Advantages	Main Limitations	Reference
Linear/Soluble Polymers	Amine, carboxyl, phosphino groups on PEG, poly(acrylamide), polystyrene backbones, and sulfonic acid	RAFT, ATRP, free-radical polymerization, and post-functionalization	Homogeneous-like selectivity, tunable solubility, and facile ligand design	Difficult recovery, limited recyclability, and low thermal stability	[35,36,37]
Crosslinked Networks & Resins	Sulfonic acid, amino, imidazolium, pyridinium on DVB-crosslinked matrices	Suspension or bulk radical polymerization with DVB crosslinker	Mechanical robustness, column/batch compatibility, and commercial availability	Lower surface area vs. POPs, and mass-transfer limitations	[23,38,39,40]
POPs	Lewis acid/base sites, boronic acid, amine, urea, metalloporphyrins, ionic liquids, and sulfonic acid	Friedel-Crafts hypercrosslinking, Sonogashira/Suzuki coupling, and Schiff-base condensation	Ultra-high BET (500–3000 m^2^ g^−1^), confinement effects, and gas adsorption	Amorphous structure, batch-to-batch variability, and processability challenges	[41,42]
Polymer–Metal Hybrids	Phosphino, amino, NHC, bipyridyl chelating groups, metalloporphyrins, ionic polymer–metal clusters, and sulfonic acid	Grafting, encapsulation, or in situ nanoparticle formation	Synergistic metal-polymer effects, enhanced stability vs. homogeneous analogues, and tunable loading	Metal leaching, characterization complexity, and precious-metal cost	[43,44]

## 3. Polymer Design Principles Governing Catalytic Performance

Beyond catalyst classification, understanding how specific polymer structural parameters translate into CO_2_ capture capacity, activation efficiency, and conversion selectivity is essential for rational catalyst design and development of these catalysts. The following subsections examine how these structural and chemical variables govern sorption and catalytic performance [17,19].

### 3.1. Role of Polymer Backbone and Functional Group Chemistry

The chemical nature of the polymer backbone establishes the baseline for CO_2_ affinity through nonspecific physisorption interactions. For example, in CTFs and CMPS, nitrogen-rich aromatic backbones based on carbazole or triphenylamine, and azo-linked polymers, provide Lewis basic N sites that interact with the Lewis acidic CO_2_ molecule, contributing to elevated CO_2_ uptake even before amine post-functionalization [28,45]. Moreover, the choice of pendant functional group and solvent is critical. Because the primary amines chemisorb CO_2_ as carbamates in nonpolar solvents and carbamic acid adducts in protic solvents, often involving two amine groups per CO_2_ molecule. Guanidine-containing sites can also promote CO_2_ binding through acid–base interactions, whereas protonated guanidinium groups primarily stabilize anionic CO_2_-derived species through electrostatic and H_2_-bonding interactions [24,46,47,48].

### 3.2. Active Site Density, Isolation, and Cooperativity

For CO_2_ capture, high amine loading (expressed as mmol N g^−1^) generally correlates with high CO_2_ uptake capacity; however, excessive amine density can lead to pore blocking, restricted CO_2_ diffusion, and reduced working capacity owing to the incomplete regeneration of densely packed carbamate species [49]. For catalytic CO_2_ cycloaddition, the proximity between the Lewis acidic metal site and the CO_2_-activating Lewis basic site is critical. Commonly, the Lewis acidic site helps in epoxide activation, or sometimes the halide functional group is involved in nucleophilic ring opening of epoxide, and Lewis basic or CO_2_-philic sites help in local enrichment or activation of CO_2_. When both sites are confined within the same micropore at a high local density, synergistic activation accelerates the rate-determining epoxide ring-opening step by 3–5-fold compared to non-cooperative analogs [50,51]. This cooperativity principle has been deliberately engineered in dual-functional POPs, where amine groups activate the epoxide through nucleophilic ring opening, followed by CO_2_ insertion into the resulting alkoxide and subsequent intramolecular cyclization to form the corresponding products [1,52].

### 3.3. Polymer Microenvironment, Pore Confinement, and Mass Transport

The polymer microenvironment surrounding each CO_2_-active site, defined by the neighboring functional groups, backbone polarity, pore size, and local electrostatic potential, profoundly influences both the thermodynamics of CO_2_ binding and the kinetics of substrate transport and activation. Ionic liquid-functionalized POPs create a CO_2_-philic local environment within their micropores that concentrates CO_2_ well above the bulk gas phase, effectively creating a high CO_2_ activity microreactor at the pore level [17]. Pore confinement also stabilizes the transition states in CO_2_ cycloaddition reactions, lowering the activation energy of the rate-determining ring-opening step [10]. The isosteric heat of CO_2_ adsorption (Q_st_), which reflects the strength of the CO_2_–sorbent interaction, can be tuned in the range of 20–45 kJ mol^−1^ through pore size engineering and functional group selection: Q_st_ values below ~30 kJ mol^−1^ favor physisorptive capture with easy thermal regeneration, while higher values (35–45 kJ mol^−1^) indicate stronger chemisorptive binding with higher working capacity at low partial pressure of CO_2_. The mass transport of CO_2_ through a polymer matrix is governed by pore connectivity and tortuosity (τ). Hierarchical macro/meso/micropore architectures minimize diffusion limitations by providing rapid gas access to the inner microporous surface, where adsorption and reactions occur at the accessible functional sites [53].

### 3.4. Hydrophilicity/Hydrophobicity and Water Tolerance

Water vapor is an omnipresent co-adsorbate in real CO_2_ capture scenarios (flue gas and direct air capture), and the water tolerance of polymer sorbents and catalysts is a critical design parameter that is often underestimated in laboratory studies using dry CO_2_. Hydrophilic amine-functionalized polymers preferentially adsorb water vapor over CO_2_ at high relative humidity (RH), which significantly reduces the working CO_2_ capacity [54]. Hydrophobic polymer matrices that incorporate fluorinated groups, long alkyl chains, or phenyl-rich backbones maintain CO_2_ uptake capacity even under humid conditions by repelling water from the pore surface while retaining CO_2_ affinity at amine or Lewis acid sites [17]. For CO_2_ cycloaddition catalysts, hydrophobic pore environments concentrate apolar epoxide substrates near the active sites while excluding moisture, which can hydrolyze epoxides and deactivate the catalyst. Thus, the optimal design balances sufficient hydrophilicity to maintain the CO_2_ chemisorption driving force with sufficient hydrophobicity to resist water competition [55].

### 3.5. Confinement, Selectivity, and CO_2_/N_2_ Separation

Pore size has a decisive influence on CO_2_/N_2_ selectivity, which is a key metric for post-combustion capture applications. CO_2_ has a kinetic diameter of 3.30 Å, whereas N_2_ is slightly larger at 3.64 Å; polymer micropores in the 4–6 Å range can exploit this difference to achieve CO_2_/N_2_ selectivities of 20–60 (Ideal Adsorbed Solution Theory (IAST, 15% CO_2_ in N_2_). Beyond size exclusion, the stronger quadrupole moment of CO_2_ relative to N_2_ results in preferential electrostatic interactions with polar functional groups (amines, triazines, and ionic liquid moieties), contributing to a thermodynamic selectivity component that operates independently of pore size. In catalytic CO_2_ cycloaddition with mixed epoxide feeds, chiral or sterically demanding pores in functionalized CMPs have been used to achieve substrate-selective ring-opening, demonstrating that confinement can provide both size selectivity and stereo-electronic differences [56,57,58].

### 3.6. Stability, Recyclability, and Leaching Resistance

Long-term sorbent and catalyst stability under repeated CO_2_ capture–release or catalytic cycling is essential for practical applications. The primary deactivation mechanisms relevant to CO_2_ applications include (i) oxidative degradation of amine groups (particularly primary amines) at elevated temperatures or in the presence of O_2_ in flue gas [59]; (ii) urea formation between adjacent amine groups at temperatures above 120 °C, which irreversibly consumes amine capacity [60]; (iii) leaching of ionic liquid or metal active sites from the polymer matrix under high-pressure CO_2_ or protic solvent conditions [61]; and (iv) hydrolytic degradation of imine, ester, or urethane linkages in the polymer backbone under humid acidic conditions [29]. Design strategies to address these mechanisms include the use of secondary or tertiary amines with higher oxidative stability, incorporation of protective alkyl or aryl substituents adjacent to the amine sites, covalent immobilization of ionic liquid units via C–C bonds rather than N-alkylation, and selection of hydrolytically stable backbone chemistries (C–C, C–N triazine, or C–N imidazolium linkages).

### 3.7. Activation Mode and Corresponding Polymer Design Requirements

Apart from polymer-centric designs, there are also different ways to synthesize polymer catalysts depends on the CO_2_ activation mode. Therefore, the three principal CO_2_ conversion activation modes such as thermochemical, photocatalytic, and electrochemical can impose fundamentally distinct polymer design requirements, which are illustrated in Table 2. As discussed, thermochemical routes (Section 4.1, Section 4.2, Section 4.3 and Section 4.4) require thermal backbone stability, high CO_2_ uptake capacity, and Lewis acid–base cooperativity at 60–200 °C and 0.1–6.0 MPa pressure. On the other hand, photocatalytic routes require extended π-conjugation with a visible-light bandgap (2.0–3.0 eV), donor–acceptor architecture for charge separation, and photochemically stable C–C or C–N linkages. Finally, electrochemical routes require electrochemically inert backbones under cathodic potentials (−0.5 to −1.5 V vs. RHE), ionic conductivity in aqueous electrolytes, and a preference for N-doped porous carbon derived from POPs or CTFs [12,25,26].

**Table 2:** Comparison of polymer design requirements across three CO_2_ conversion activation modes.

Design Parameter	Thermochemical	Photocatalytic	Electrochemical
Energy input	Heat (60–200 °C, 0.1–6.0 MPa)	Photons (λ > 420 nm, 1 bar)	Electricity (−0.5 to −1.5 V vs. RHE)
Key polymer property	Thermal stability; Lewis acid–base cooperativity	Bandgap 2.0–3.0 eV; D–A architecture	Electronic conductivity; electrochemical inertness
Preferred backbone	HCPs, CTFs, crosslinked resins	CMPs (carbazole, triazine, porphyrin)	N-doped porous carbons from POPs/CTFs
Critical stability concern	Thermal/hydrothermal degradation	Photodegradation of ester/imine linkages	Reduction of labile backbone groups

## 4. Functional Polymer Catalysts for CO_2_ Capture and Conversion

CO_2_ capture and conversion, commonly known as Reactive CO_2_ Capture (RCC), has emerged as a sustainable strategy for mitigating CO_2_ emissions from industrial and other anthropogenic sources by transforming them into value-added chemicals and fuels. This integrated approach not only reduces carbon emissions but also enables the utilization of CO_2_ as a renewable carbon feedstock for synthesizing functional materials, chemical intermediates, and energy-related products. Various valuable compounds, including 2-oxazolidinones, organic carbamates, CCs, methane, methanol, formate, ethylene, ethanol, acetic acid, and polymers, can be produced via CO_2_ conversion processes [56,62,63,64]. In recent years, functional polymer catalysts have attracted significant interest in RCC applications owing to their tunable surface chemistry, porosity, structural versatility, thermal stability, and excellent catalytic efficiency in CO_2_ capture and conversion processes. These materials provide effective platforms for the transformation of captured CO_2_ into useful products via various catalytic pathways [42,65]. The catalytic valorization of CO_2_, which involves converting this greenhouse gas into useful chemicals and fuels using functional polymer catalysts, is a central objective of sustainable chemistry. Functional polymer catalysts offer distinct advantages in CO_2_ chemistry, including high CO_2_ uptake capacity, tunable basicity for activation, and stability under the pressures and temperatures required for conversion. This section constitutes the core of the review, covering the full spectrum of CO_2_ capture and conversion pathways enabled by functional polymer-based catalysts. The quantitative performance data are presented in Table 3, Table 4, Table 5 and Table 6. The discussion proceeds from CO_2_ capture by adsorption through CO_2_ cycloaddition chemistry and CO_2_ hydrogenation to integrated dual-functional systems that perform capture and conversion in a single step.

### 4.1. CO_2_ Capture: Adsorption by Functional Polymer Sorbents

It is important to distinguish between CO_2_ adsorption, where the polymer serves as a sorbent for capture and concentration, and catalytic activation/conversion, where the polymer directly participates in the conversion of CO_2_ to a product. For example, amine-functionalized POPs (polyethylenimine-grafted HCPs and poly(allylamine)-impregnated frameworks) achieve uptake capacities of 2–4 mmol CO_2_ g^−1^ at 0.15 bar via chemisorption through the formation of carbamate and carbamic acid. The regenerability of amine sorbents under mild heating (90–120 °C) allows for cyclic operation without significant capacity loss over 50 or more cycles [66,67]. However, a rigorous cross-study comparison of CO_2_ uptake capacities is constrained by the heterogeneous testing conditions reported in the primary literature (varying temperature, pressure, moisture content, and amine loading), which is a known limitation of the field.

**Table 3:** Representative functional polymer catalysts for CO_2_ capture: performance metrics and recyclability.

Polymer Catalyst/Sorbent	Surface Area	Reaction Conditions	Performance Metric (CO_2_ Uptake in mmol g^−1^ or mg)	Recyclability (Number of Cycles/Time)	Reference
PEI70@PGD-H (PGD = poly(glycidyl methacrylate–divinylbenzene and 70 = Mass % of PEI)	82.5 m^2^ g^−1^	At 1 bar CO_2_ and 323 K	4.3 mmol g^−1^	20 cycles at 1 bar and 323 K	[68]
		At 0.15 bar CO_2_ and 323 K	3.0 mmol g^−1^		
Oxygen-doped hexaazatriphenylene-based CTFs (O-doped HAT-CTFs-450/600)	1090 m^2^ g^−1^	At 1 bar CO_2_ and 297 K	4.8 mmol g^−1^	5 cycles at 0.15 bar and 297 K	[69]
		At 0.15 bar CO_2_ and 297 K	2.0 mmol g^−1^		
		At 1 bar CO_2_ and 273 K	6.3 mmol g^−1^		
		At 0.15 bar CO_2_ and 273 K	3.0 mmol g^−1^		
Benzyl alcohol (BA)-based hypercrosslinked microporous polymers (BA-HCMPs)	1101 m^2^ g^−1^	At 1 bar CO_2_ and 273 K	3.03 mmol g^−1^	Not reported	[70]
1,3-diyne-linked CMP (LKK-CMP-1)	467 m^2^ g^−1^	At 1 bar CO_2_ and 273 K	9.78 wt %	4 cycles at 1 bar and 298 K	[57]
PEI-grafted-mesocellular silica foam (MCF@G-35.6% PEI)	127.74 m^2^ g^−1^	At 1 bar CO_2_, 323 K, and 50% RH	2.78 mmol g^−1^	50 cycles at 1 bar, 323 K, and 0% RH	[66]
PEI-crosslinked cellulose (PCC-1) aerogel sorbent	234.2 m^2^ g^−1^	At 298 K and pure dry CO_2_ (200 mL/min)	2.31 mmol g^−1^	10 cycles at 1 bar and 313 K	[71]
Benzyl chloride-based HCP (B-Cl)	1248.869 m^2^ g^−1^	At 1 bar CO_2_ and 273 K	75.14 mg g^−1^	5 cycles at 1 bar and 273 K	[72]
In situ polymerized 2-methyl-2-oxazoline on SiO_2_	94.6 m^2^ g^−1^	At 298 K, 65% RH, and 400 ppm of CO_2_	1.50 mmol g^−1^	10 cycles at 298 K, 25% RH, and 400 ppm of CO_2_	[73]
Quaternary ammonium-modified ionic lignin polymer	Not reported	Concentrated CO_2_ source and 298 K	1.06 mmol g^−1^	4 cycles at 298 K from concentrated CO_2_ source	[74]
Hydroxy-functionalized porous aromatic framework (PAF-32-OH)	1608 m^2^ g^−1^	At 1 bar CO_2_ and 273 K	2.27 mmol g^−1^	Not reported	[75]
Pyromellitic dianhydride & triazine-based porous polyimide (TPI) polymer network (TPI-1)	809 m^2^ g^−1^	At 1 bar CO_2_ and 273 K	2.45 mmol g^−1^	Not reported	[76]

Zhu et al. [69] developed a novel, highly stable, and ultramicroporous functionalized POP, namely HAT-CTFs, for efficient CO_2_ capture using an in situ ZnCl_2_-catalyzed two-step calcination strategy (Figure 1a). The synthesized framework exhibited a high surface area of 1090 m^2^ g^−1^, ultra-micropores smaller than 0.6 nm, and abundant O-doped CO_2_-philic surface sites. These structural characteristics significantly enhanced the adsorption performance, enabling CO_2_ uptake capacities of 6.3 and 4.8 mmol g^−1^ at 273 and 297 K, respectively, under 1 bar pressure, as shown in Figure 1b. Even at a lower pressure of 0.15 bar, the material maintained considerable CO_2_ adsorption capacities of 3.0 and 2.0 mmol g^−1^ at 273 K and 297 K, respectively (Table 3). These results demonstrate the critical role of ultramicro-porosity and heteroatom doping in improving CO_2_ capture efficiency [42,69,77]. Similarly, Zhang et al. [70] reported the fabrication of BA-derived HCMPs enriched with surface hydroxyl functionalities using a simple FeCl_3_-catalyzed Friedel–Crafts alkylation process. The resulting polymer displayed a BET surface area of 1101 m^2^ g^−1^ and showed effective CO_2_/N_2_ separation with a selectivity value of 42, which was attributed to its hierarchical pore architecture consisting of abundant mesopores and dominant micropores. As discussed in Table 3, the optimized material achieved a notable CO_2_ adsorption capacity of 3.03 mmol g^−1^ at 273 K under 1 bar pressure, demonstrating the effectiveness of hydroxyl-functionalized porous structures for selective gas capture [70].

In another study, Ghorai and Chung [74] introduced an innovative water-soluble lignin-based ionic polymer sorbent derived from biomass for CO_2_ capture. In this study, lignin was transformed into a functional CO_2_-capturing material through a facile chemical modification using glycidyl trimethylammonium chloride (GTAC), as shown in Figure 1c. The lignin precursor contained a total hydroxyl group content of 4.48 mmol g^−1^. Functionalization was carried out by dissolving lignin in 0.5 M NaOH solution, followed by a reaction with GTAC at 65 °C for 5 h and subsequent overnight stirring at room temperature. The product was then purified by dialysis in H_2_O for 72 h and freeze-dried to obtain the final polymer product. The resulting material contained covalently grafted quaternary ammonium groups, which served as active sites for CO_2_ capture via bicarbonate formation through chemisorption, as confirmed by ^13^C-NMR analysis (Figure 1d,e). The developed sorbent exhibited a total CO_2_ uptake capacity of 1.10 mmol g^−1^ under concentrated CO_2_ conditions, consisting of equal contributions from chemisorption (0.55 mmol g^−1^) through surface amine functionalities and physisorption (0.55 mmol g^−1^) on the catalyst surface, as shown in Figure 1f and Figure 1g, respectively. Importantly, the captured CO_2_ could be easily released by mild heating at 60 °C, and the catalyst maintained a stable performance over repeated adsorption–desorption cycles, demonstrating excellent recyclability. To assess its practical applicability, the material was evaluated for direct air capture and exhibited an adsorption capacity of 0.60 mmol g^−1^. Overall, these studies demonstrate that newly designed biomass-derived and functionalized porous polymer catalysts offer significant promise for CO_2_ capture and utilization applications. Their tunable surface chemistry, structural robustness, recyclability, and environmentally benign nature render them highly attractive candidates for carbon capture and conversion applications, as well as for broader catalytic and adsorption-related technologies [74].

### 4.2. Cycloaddition of CO_2_ with Epoxides: Cyclic Carbonate Synthesis

The cycloaddition of CO_2_ with epoxides has emerged as one of the most promising approaches in sustainable organic chemistry for the effective utilization of CO_2_ as an abundant, renewable, and low-cost C1 feedstock. This reaction enables the direct conversion of CO_2_ into CCs, a class of highly value-added compounds with extensive applications as electrolytes in batteries, solvents, pharmaceutical intermediates, and precursors for polymer synthesis. Recent progress in catalyst development, including metal-based complexes, ionic liquids, functional polymer catalysts, and porous materials, has substantially enhanced the efficiency and practicality of these transformations. These catalytic systems have enabled improved CO_2_ adsorption and activation, faster reaction kinetics, and greater selectivity toward CC formation. Functional polymer catalysts have attracted attention for the conversion of epoxides into CCs by incorporating CO_2_ because of their high surface areas, easy and tunable functionalization, and porous, metal-free structures [56,65,78,79].

**Table 4:** Representative functional polymer catalysts for the cycloaddition of CO_2_ and epoxides to CCs: performance metrics and their recyclability.

Polymer Catalyst/Sorbent	Surface Area	Epoxide (Concentration)	Reaction Conditions	Performance Metric (TON or TOF (h^−1^) and Conversion or Yield)	Recyclability (Number of Cycles/Time in Hours)	Reference
Fe-Hydroxyl-assisted ionic bifunctional HCPs (HCPs-HQ-Fe)	820 m^2^ g^−1^	1,2-epoxybutane (41.6 mmol)	100 °C, 0.1 MPa CO_2_, and 20 h	3241 and 99% yield	6 cycles	[80]
Zn-HUT4	76 m^2^ g^−1^	Propylene oxide (PO, 71.55 mmol)	110 °C, 2 MPa CO_2_, 1 h, and TBAB (0.27 mmol)	6812 h^–1^ and >99% conversion	5 cycles	[81]
P(MBA-V_3_Br)-2 (MBA = *N,N′*-Methylene bisacrylamide and V_3_Br = Multi-vinylimidazolium bromide monomer)	13.1 m^2^ g^−1^	Epichlorohydrin (ECH, 40 mmol)	80 °C, 0.1 MPa CO_2_, and 12 h	Not reported and 95% yield	10 cycles	[82]
Mesoporous melamine-formaldehyde resin (MMFR)	328 m^2^ g^−1^	ECH (20 mmol)	120 °C, 2 MPa of CO_2_, and 4 h	1884 h^–1^ and 96% conversion	109 h	[83]
MMFR	328 m^2^ g^−1^	ECH (20 mmol)	140 °C, 2 MPa of CO_2_, and 2 h	7918 h^–1^ and >99% conversion	Not reported	[83]
Mesoporous melamine-formaldehyde resin synthesized at 250 °C (MMFR250)	290 m^2^ g^−1^	ECH (20 mmol)	120 °C, 2 MPa of CO_2_, and 4 h	2147 h^–1^ and 80% conversion	Not reported	[83]
Hydroxylamine-anchored covalent aromatic polymer (CAP) functionalized with 1,3-diamino-2-propanol (CAP-DAP)	517 m^2^ g^−1^	ECH (20 mmol)	60 °C, 0.1 MPa of CO_2_, 12 h, and TBAB (0.4 mmol)	Not reported and 98% conversion	6 cycles	[84]
Amine-anchored covalent triazine aromatic polymers based on biphenyl (CTP-1-NH_2_)	875 m^2^ g^−1^	ECH (20 mmol)	40 °C, 0.1 MPa of CO_2_, 36 h, and TBAB (2 mol%)	Not reported and 93% conversion	6 cycles	[85]
Amine-anchored covalent triazine aromatic polymers based on 1,3,5-triphenylbenzene (CTP-2-NH_2_)	1387 m^2^ g^−1^	ECH (20 mmol)	40 °C, 0.1 MPa of CO_2_, 36 h, and TBAB (2 mol%)	Not reported and 91% conversion	Not reported	[85]
Zinc(II) porphyrin-based porous ionic polymers based on divinyl benzene (PIP-ZnTIPP/DVB)	357 m^2^ g^−1^	ECH (10 mmol)	120 °C, 1 MPa of CO_2_, and 6 h	759 h^−1^ and 99% yield	6 cycles	[86]
Aluminum–porphyrin-based PIP (AlPor-PIP-Br)	761 cm^2^ g^−1^	ECH (3 mmol)	100 °C, 1 MPa of CO_2_, and 4 h	1125 h^−1^ and 90% yield	6 cycles	[87]
CoTPP-PiP(Br)	116.2 m^2^ g^−1^	PO (3 mmol)	100 °C, 1 MPa of CO_2_, and 10 h	Not reported and >99% conversion	8 cycles	[88]
PIL-Br_1.0_@Zn-CTM (PIL = poly(ionic liquid) and Zn-CTM = Zn/N co-doped carbon material)	92 m^2^ g^−1^	ECH (5 mmol)	100 °C, 1 MPa of CO_2_, and 13 h	723 and 94% yield	7 cycles	[89]
1-(2-Hydroxyl-ethyl)-imidazolium-based ionic liquids (HEIMBr) anchored on highly cross-linked polystyrene resin (PS) (PS-HEIMBr)	Not reported	PO (0.1 mol)	120 °C, 2.5 MPa of CO_2_, and 4 h	Not reported and 99% conversion	6 cycles	[90]
Co-CMP	965 m^2^ g^−1^	PO (25 mmol)	100 °C, 3 MPa of CO_2_, 1 h, and TBAB (1.8 mmol)	201 and 98.1% yield	22 cycles	[58]

Zhou et al. reported the development of a novel heterogeneous catalyst, a Zn-anchored ionic porous organic polymer (Zn-iPOP) designated as Zn-HUT4, synthesized through a simple and cost-effective method [81]. The prepared catalyst exhibited a porous architecture with a BET surface area of 76 m^2^ g^−1^ and an average pore size of 2.2 nm, which is important for efficient catalytic performance. The synthesized Zn-HUT4 was successfully employed in the cycloaddition of CO_2_ with propylene oxide and various other epoxides to produce CCs [81]. The catalyst demonstrated excellent catalytic performance, delivering high conversion and selectivity, both exceeding 99%, for the synthesis of a wide range of CC products at 110 °C. In addition, Zn-HUT4 exhibited remarkable structural stability and could be efficiently recycled for up to five consecutive reaction cycles without a significant loss of activity. The superior catalytic performance of Zn-HUT4 was associated with its CO_2_ adsorption capability, with uptake capacities of 3.4 and 2.5 cm^3^ g^−1^ at 273 and 298 K, respectively. This enhanced adsorption behaviour arises from the presence of polar functional groups containing N and O atoms within the HUT4 framework, which promote strong interactions with CO_2_ molecules. Initially, the catalytic mechanism involves coordination of the oxygen atom of the epoxide with the Zn(II) centers of Zn-HUT4, resulting in the polarization of the C–O bond. Subsequently, Br^−^ ions from tetrabutylammonium bromide attack the epoxide carbon, facilitating ring opening through the cooperative action of Br^−^ and Zn^2+^. The generated alkoxide intermediate then reacts with CO_2_ to form a carbonate intermediate, which undergoes intramolecular cyclization to yield the corresponding CC product [81].

Bui et al. developed low-cost functional polymer catalysts, namely MMFR and MMFR250, through a simple hydrothermal synthesis route without the use of organic solvents or templates. The MMFR250 catalyst was obtained by further thermal treatment of MMFR at 250 °C for 5 h under a N_2_ atmosphere. The synthesized mesoporous catalysts exhibited high surface areas of 328 and 290 m^2^ g^−1^ for MMFR and MMFR250, respectively, with average pore sizes of 24.2 and 24.3 nm, respectively. These materials also demonstrated excellent CO_2_ adsorption capacities at 298 K, reaching 5.8 mmol g^−1^ for MMFR and 2.78 mmol g^−1^ for MMFR250, respectively. The catalytic performance of these materials was evaluated for the cycloaddition of gaseous CO_2_ with various epoxides, including styrene oxide, butylene oxide, and epichlorohydrin, under continuous-flow reaction conditions to synthesize CCs. The MMFR catalyst exhibited outstanding catalytic activity, achieving over 99% conversion and 99% selectivity at 140 °C, with a high TOF of 7918 h^−1^ (Table 3). Meanwhile, MMFR250 delivered 80% conversion with selectivity exceeding 99% at 120 °C, accompanied by a TOF of 2147 h^−1^ (Table 3). The excellent catalytic performance of these materials is mainly attributed to their abundant surface basic sites and strong CO_2_ adsorption characteristics of these materials. CO_2_-Temperature Programmed Desorption (CO_2_-TPD) analysis revealed basic site densities of 172 and 56 μmol g^−1^, along with CO_2_ desorption activation energies of 92.1 and 64.5 kJ mol^−1^ for MMFR and MMFR250, respectively. The basicity of these catalysts arises from the nucleophilic amine functionalities present within the resin framework. These amine sites act as active centers for the simultaneous activation of both CO_2_ and epoxide substrates, thereby facilitating efficient cycloaddition and the formation of the corresponding CC products [83].

CMPs are a large and versatile family of porous organic frameworks that integrate permanent microporosity with extended π-electron conjugation. Owing to their high surface areas, tunable architectures, robust chemical stability, and ability to host diverse metal active sites, enabling efficient mass transfer, CMPs have attracted considerable attention for applications in CO_2_ capture and utilization for enhanced catalytic activities [29].

Yong et al. developed metal-based CMPs such as Co-CMP and Al-CMP materials for cycloaddition of CO_2_ with epoxides to produce CCs. As shown in Figure 2a (method A), Co-CMP was constructed via a Sonogashira coupling reaction between 1,3,5-triethynylbenzene and a cobalt-salen complex under inert conditions. After polymerization, the material was purified by solvent washing and Soxhlet extraction several times, producing a porous Co-CMP. Similarly, for comparison, a metal-free CMP was synthesized using the related salen ligand, while the post-synthetic introduction of ‘Al’ species into the CMP afforded Al-CMP, as represented in method B. Both Co-CMP and Al-CMP materials synthesized were insoluble porous polymers and possessed high chemical stability and high surface areas (965 and 798 m^2^ g^−1^). As displayed in Figure 2b–e, morphological analysis revealed that both Co-CMP and Al-CMP materials comprised comparatively uniform sub-micrometer spherical particles. In Figure 2b,d, the SEM images represent densely packed spherical morphologies, whereas in Figure 2c,e, the HR-TEM images reveal the presence of ultra-micropores with dimensions of approximately 0.5 nm. The porous framework provides many readily available active sites and improves the adsorption and movement of reactants, making these materials suitable heterogeneous catalysts for CO_2_ conversion. The catalytic capabilities of Co-CMP and Al-CMP were investigated for the cycloaddition of PO with CO_2_ to generate the respective CC. Under benign reaction conditions, as shown in Figure 2f, Co-CMP delivered the highest activity with TBAB as the cocatalyst, exceeding that of the corresponding homogeneous Co-salen catalyst. This enhanced performance was attributed to the porous framework, which enhanced the CO_2_ concentration around the catalytic sites and facilitated reactant activation. Al-CMP also represented high activity under the same reaction conditions, even though its efficiency was comparatively lower than that of Co-CMP. Furthermore, a comparative study of the catalytic performance of conventional catalysts for this reaction, such as KI and KI/β-cyclodextrin, highlighted the dominance of the synthesized CMP-based materials. As shown in Figure 2f, while the industrial benchmark catalysts and the TBAB-only system displayed insufficient activity under ambient conditions, Co-CMP and Al-CMP achieved significantly higher propylene carbonate (PC) yields with TONs of 167 and 160, respectively. The catalytic performance with respect to the yields followed the order: Co-CMP (81.5) > Al-CMP (78.2) > Salen-Co-OAc (77.1) >> TBAB (20.4) > KI/β-CD (3.9) ≈ KI (3.8).

Moreover, the catalytic superiority of the Co-CMP material was further supported at elevated temperatures (i.e., 100 °C) and CO_2_ pressures (i.e., 3.0 MPa). Under these conditions, both materials exhibited substantially higher PC yields than the corresponding homogeneous catalyst and TBAB-only system. As shown in Figure 2g, the catalyst stability was investigated through repeated recycling experiments. Co-CMP exhibited excellent durability and maintained nearly constant activity over numerous catalytic cycles under high-pressure (i.e., 3.0 MPa) and high-temperature (i.e., 100 °C) conditions. Although gradual deactivation was observed after extended operation under ambient conditions (i.e., 25 °C and 0.1 MPa), the loss of activity was mainly associated with partial ‘Co’ leaching from the CMP framework. Importantly, the catalytic performance was fully restored through simple ‘Co’ reloading, confirming the robustness and regenerability of the polymer support. In contrast, Al-CMP exhibited lower stability. A significant decline in performance was observed after only a few reaction cycles, which was ascribed to the hygroscopic nature of the Al-salen centers, which are prone to deactivation through contact with trace amounts of H_2_O. The reactivity of Al-CMP to moisture is steady, with reduced catalytic performance in water-containing reaction systems. Finally, the synergistic effects of the metal-salen Lewis acid sites and TBAB-derived bromide ions, combined with the hierarchical porous CMP framework, could activate the CO_2_ and epoxide substrates and promote efficient CC formation, which is responsible for the excellent catalytic performance of Co-CMP and Al-CMP. Notably, these materials represent rare examples of heterogeneous catalysts capable of promoting CO_2_-epoxide cycloaddition under both atmospheric pressure and room temperature. The outstanding catalytic performance of Co-CMP was also reflected in large-scale experiments, in which very high TONs were achieved during prolonged reaction periods. These results demonstrate the practical potential of porous metallosalen-based CMPs as catalysts for CO_2_ utilization [58].

Ravi et al. developed a unique covalent organic polymer (COP) called hydroxylamine-anchored CAP-DAP through a straightforward synthetic approach using 1,3,5-benzene tricarbonyl chloride and p-terphenyl, followed by functionalization with 1,3-diamino-2-propanol (DAP). The successful incorporation of DAP into the CAP framework was confirmed by FTIR and XPS analyses, which revealed characteristic peaks corresponding to primary and secondary amine functionalities and hydroxyl(–OH) groups. The synthesized functional polymer catalyst exhibited a high surface area of 517 m^2^ g^−1^, with an average pore size of 1.31 nm. The catalytic performance of the catalyst was evaluated for the cycloaddition of epichlorohydrin with CO_2_ for the synthesis of cyclic carbonates. The catalyst demonstrated excellent activity, achieving 97% conversion and 99% selectivity toward epichlorohydrin carbonate at a relatively low temperature of 60 °C. When the reaction temperature was increased to 80 °C, complete conversion was obtained. The high catalytic activity and recyclability were attributed to the cooperative action of the hydroxyl and amine functional groups within the polymer framework. Initially, these groups activated the epoxide through H_2_-bonding interactions, facilitating Br^−^-induced ring opening. The resulting alkoxide intermediate then reacts with the adsorbed CO_2_ to form a carbonate intermediate, which subsequently undergoes intramolecular cyclization to generate the CC product and regenerate the catalyst [84].

Lei et al. reported the synthesis of dual hydroxyls-functionalized polymeric phosphonium bromide (PQPBr-2OH) through a straightforward post-functionalization strategy. First, poly(tris(4-vinylphenyl)phosphine) (POL-PPh_3_) was reacted with epibromohydrin to produce an epoxy-functionalized phosphonium polymer (PQPBr). Subsequently, hydrolytic ring opening of the epoxy groups in hot water produced the dual-hydroxyl-functionalized porous ionic polymer, PQPBr-2OH, as shown in Figure 3a. The resulting material possessed a high surface area, large pore volume, and hierarchical pore architecture, making it an attractive metal-free heterogeneous catalyst for the cycloaddition of CO_2_ with epoxides. Nitrogen adsorption–desorption measurements (Figure 3b) revealed that both PQPBr and PQPBr-2OH expressed mixed type I and type IV isotherms, indicating the presence of micro- and mesoporous structures. A pronounced uptake at low relative pressures confirmed the abundance of micropores, whereas increased adsorption at higher relative pressures suggested the presence of larger pores in the carbon framework. The hysteresis loop observed at high relative pressures further indicates the existence of mesoporosity. The pore size distribution analysis demonstrated that PQPBr-2OH contained a hierarchical network composed predominantly of micropores and mesopores, together with a limited fraction of macropores, as shown in the inset of Figure 3b. Such a porous architecture is advantageous for heterogeneous catalysis because it enhances both the accessibility of the active sites and the diffusion of the reactants and products. FT-IR spectroscopy revealed the effective incorporation of -OH functionalities. PQPBr highlighted a characteristic epoxy group vibration near 1270 cm^−1^, which faded after the hydrolysis step. Concurrently, a new band related to the -OH groups appeared at approximately 1045 cm^−1^, confirming the production of PQPBr-2OH. Structural evaluation was also conducted using solid-state ^13^C-NMR, solid-state ^31^P-NMR, and XPS spectroscopy. As displayed in Figure 3c, the ^13^C-NMR spectra depict the signals linked to aromatic carbon atoms and aliphatic carbon chains, which are consistent with the polymer network. The ^31^P-NMR spectra shown in Figure 3d reveal a major resonance ascribed to the phosphonium species and a tiny signal emerging from the leftover unreacted phosphine groups, indicating efficient phosphonium functionalization. XPS measurements, as shown in Figure 3e, further verified the presence of C, O, Br, and P elements. As illustrated in Figure 3f, the increased O 1s signal intensity observed for PQPBr-2OH compared with that of PQPBr provides supporting evidence for hydroxyl introduction. Furthermore, the feature of the P 2p signal was related to phosphonium salt formation, validating the effective synthesis of the bifunctional porous ionic polymer.

The catalytic performance of PQPBr-2OH was assessed for the cycloaddition of epoxides with CO_2_ to produce CCs. Owing to its large surface area and multilevel porosity, the PQPBr-2OH catalyst exhibited significant catalytic activity under mild conditions. The combination of available active sites and effective mass transport considerably assisted its activity. The endurance of the PQPBr-2OH catalyst was investigated for 10 consecutive runs using styrene oxide as a model substrate, as shown in Figure 3h. Regardless of the reaction scale, when increased tenfold, PQPBr-2OH preserved outstanding catalytic performance, furnishing nearly the same CC yields. To maintain the catalyst stability after each experiment, the catalyst was separated, washed, dried, and reused directly without post-treatment. Mechanistic investigations indicated that the hydroxyl groups played a vital role in facilitating the cycloaddition reaction. Catalysts comprising hydroxyl functionalities demonstrated considerably higher activity than similar materials without these groups. Previous studies have established that epoxide ring opening is the rate-determining step in this reaction. In PQPBr-2OH, the bromide anion serves as the nucleophilic species responsible for attacking the epoxide ring, whereas the hydroxyl groups facilitate substrate activation through H_2_-bond interactions. This cooperative activation reduces the activation barrier for ring-opening and accelerates the overall reaction. As illustrated in Figure 3i, credible catalytic mechanism starts with the development a H_2_ bond between a hydroxyl group of PQPBr-2OH and the oxygen atom of the epoxide. Next, the bromide anion attacks the less sterically hindered carbon atom of the epoxide, generating a haloalkoxide intermediate. This intermediate reacts with CO_2_ to generate an alkyl carbonate species, which eventually undergoes intramolecular cyclization to produce the corresponding CC and restore the catalytic site. The synergistic action of hydroxyl groups and bromide anions is therefore accountable for the outstanding catalytic efficiency witnessed for PQPBr-2OH [91].

In another study, Liu et al. developed a composite functional polymer catalyst based on poly(ionic liquid)s using a multistep synthetic strategy. Initially, a Zn-CTM was prepared by calcining a precursor mixture containing the ionic liquid [Bmim][NTf_2_], the organic monomer 2,4,6-tris(4-formylphenoxy)-1,3,5-triazine (TFPT), melamine, and the metal salt zinc acetate dihydrate. In the second stage, the ionic liquid monomer, 1,4-bis(3-vinylimidazole)butane dibromide (C_4_ImBr), was synthesized using a solvothermal method. Finally, PIL-Br_x_@Zn-CTM functional polymer nanocomposites (x = 0.5, 1.0, and 1.5) were fabricated via in situ free-radical polymerization of vinyl-functionalized C_4_ImBr monomers on the Zn-CTM surface. Among the synthesized materials, PIL-Br_1.0_@Zn-CTM exhibited a BET surface area of 91 m^2^ g^−1^ and a pore size of 3.9 nm. In comparison, the pristine Zn-CTM displayed a significantly higher surface area of 622 m^2^ g^−1^ with a broader pore size distribution ranging from 3.9 to 17.5 nm in diameter. The reduction in the surface area after polymer incorporation was attributed to random polymer growth on the Zn-CTM surface, which partially blocked or narrowed the accessible pores. A similar trend was observed for the CO_2_ adsorption performance. The bare Zn-CTM showed the highest CO_2_ uptake capacity of 2.3 mmol g^−1^ at 273 K, whereas the PIL-modified nanocomposite exhibited a lower uptake of 1.2 mmol g^−1^ under the same conditions. This behaviour correlates with the reduction in surface area and the altered distribution of Lewis basic adsorption sites following polymer deposition. Despite this decrease in adsorption capacity, the catalytic performance of the composite materials was impressive. Catalytic evaluation was performed under simulated flue gas conditions consisting of 15% CO_2_ and 85% N_2_, using epichlorohydrin as the epoxide substrate under additive-free conditions [89].

The PIL-Br_1.0_@Zn-CTM catalyst demonstrated outstanding activity, achieving 99% selectivity, an 87% product yield, and a TON of 669 for CC formation at 100 °C and 1.0 MPa pressure under solvent-free conditions. The product yield was found to depend strongly on the steric hindrance of the epoxide substrate, where epoxides bearing chloromethyl (–CH_2_Cl), bromomethyl (–CH_2_Br), and hydroxymethyl (–CH_2_OH) groups showed excellent CC yields owing to their electron-withdrawing characteristics and low steric hindrance, which enhanced Br^−^-mediated ring-opening in the PIL-Br_1.0_@Zn-CTM catalytic system. Meanwhile, epoxides substituted with n-butyl or phenyl groups afforded moderate to good yields owing to their relatively high steric hindrance. The remarkable catalytic efficiency of PIL-Br_x_@Zn-CTM can be attributed to the synergistic presence of multiple active sites within the nanocomposite structure. These include Lewis basic sites (pyridinic-N and C–N functionalities), Lewis acidic centers (Zn^2+^), and nucleophilic bromide ions (Br^−^), which cooperatively facilitate the sequential activation of CO_2_ and epoxide substrates. The reaction proceeds via epoxide activation (I), nucleophilic ring opening (II), CO_2_ insertion (III), and subsequent intramolecular cyclization to yield the corresponding CC (IV), simultaneously restoring the original catalyst. Importantly, the functional nanocomposite catalyst exhibited excellent structural stability and recyclability, maintaining its catalytic performance over seven consecutive cycles. These findings demonstrate the significant potential of multifunctional polymer-based nanocomposites as integrated materials for efficient RCC applications [89].

Generally, the cycloaddition of CO_2_ with epoxides to produce CCs involves a common proposed sequence of mechanistic steps, including epoxide activation, nucleophilic ring opening, CO_2_ insertion, and intramolecular cyclization. Growing evidence reveals that the structure and architecture of the polymer catalyst can significantly influence the energetics and kinetics of each mechanistic step. In addition, mechanistic and computational investigations have identified epoxide ring opening as the most energetically demanding step, making it a critical factor in overall performance. Furthermore, the catalytic performance of POPs is controlled by the spatial arrangement of active centers, active site accessibility, nature of cooperative functionalities, pore architecture, reactant diffusion, and local CO_2_ concentration. For example, recent studies on ionic hyper-crosslinked polymers further demonstrated that the incorporation of H_2_-bond-donating functionalities such as -OH, -COOH, and -NH_2_ groups significantly lowers the associated activation barrier (0.68 eV versus 1.32 eV for hydroxyl-deficient analogs) for epoxide ring opening through cooperative activation and transition-state stabilization. Moreover, ionic hyper-crosslinked polymers promote cooperative interactions between H_2_-bond-donating functionalities and nucleophilic halides. POPs, HCPs, and CTFs increase CO_2_ adsorption and mass transport owing to their high surface areas and hierarchical pore structures. These pore-confined microenvironments facilitate the local concentration of CO_2_ near the catalytically active sites, thereby accelerating CO_2_ insertion into the alkoxide intermediate and boosting CC formation. Additionally, the rational integration of CO_2_-philic groups, Lewis basic nitrogen sites, and ionic functionalities within the polymer network enhances reactant activation and intermediate stabilization, often leading to outstanding catalytic performance compared with comparable monofunctional systems. Consequently, catalytic activity is determined not only by the nature of the active sites but also by the ability of the polymer architecture to regulate mass transport, stabilize key intermediates, and lower reaction barriers through multifunctional cooperativity [17,52,80,91,92].

### 4.3. Polymer-Supported Metal Catalysts for CO_2_ Hydrogenation

CO_2_ hydrogenation using green H_2_ has emerged as an effective strategy for reducing carbon emissions while simultaneously converting CO_2_ into valuable chemicals. The development of efficient catalysts is essential for achieving high performance and enabling large-scale industrial applications of these materials [93]. Functional polymer catalysts have attracted significant attention owing to their tunable surface functionalities, structural versatility, and excellent catalytic properties. In particular, the ability to precisely tailor the surface chemistry and porosity plays a crucial role in directing the reaction pathways and improving product selectivity [56,65].

**Table 5:** Representative functional polymer catalysts for CO_2_ hydrogenation: Performance metrics and recyclability.

Polymer Catalyst/Sorbent	Surface Area	Reaction Conditions	Performance Metric (Product, TON or TOF (h^−1^), and Yield)	Recyclability (Cycles)	Reference
p-PNP-Ir	1402 m^2^ g^−1^	High-pressure reaction kettle, 150 °C, H_2_O (5 mL), DBU (5 mmol), CO_2_ + H_2_ (20:50 bar), and 12 h	HCOOH, 585,400, and not reported	15 cycles	[94]
Pt-supported on an amine-functionalized ordered mesoporous polymer (Pt/SNOMP)	440 m^2^ g^−1^	Stainless-steel batch reactor, 120 °C, 1,4-dioxane (90 mL) and morpholine (4.86 mL) of Step 1: 120 °C, CO_2_ + H_2_ (2:2 MPa), and 48 h; Step 2: 120 °C, 4 MPa H_2_, and 24 h	CH_3_OH, 28.11 h^−1^, and 72%	10 cycles	[95]
Ir-HCP-triphenylmethane (TPM) (Ir-HCP-(TPM))	1046 m^2^ g^−1^	Parr reactor, 80 °C, 1 M KOH (8 mL), H_2_ + CO_2_ (2.5:2.5 MPa), and 4 h	HCOOH, 50,816, and 6098 µmol	10 cycles	[96]
Ir-HCP-benzene (B) (Ir-HCP-(B))	628 m^2^ g^−1^	Parr reactor, 80 °C, 1 M KOH (8 mL), H_2_ + CO_2_ (2.5:2.5 MPa), and 1 h	HCOOH, 9733, and 1168 µmol	Not reported	[96]
Ru-integrated nitrogen- and phosphorus-containing cyclophosphazene polymers (Ru/p-dop-POMs)	17.2 m^2^ g^−1^	Parr reactor, 120 °C, Et_3_N (20 mmol) and H_2_O (5 mL), H_2_ + CO_2_ (4:4 MPa), and 24 h	HCOOH, 25,400, and 3.36 M	3 cycles	[97]
Ru-MACHO- porous organometallic polymer (Ru-MACHO-POMP)	580 m^2^ g^−1^	Stainless steel reactor, 120 °C, Et_3_N (60 mmol) and H_2_O (11.6 mL), H_2_ + CO_2_ (6:2 MPa), and 3 min	HCOOH, 31,700, and 0.82 M	5 cycles	[98]
IrCl_3_-phenanthroline-functionalized POP (IrCl_3_–phen-POP)	560 m^2^ g^−1^	Stainless steel reactor, 120 °C, aqueous Et_3_N solution (1.0 M, 20.0 mL), H_2_ + CO_2_ (4:4 MPa), and 2 h	HCOOH, 14,400, and not reported	3 cycles	[99]
[RuHCl(CO)(PPh_3_)(bis-NHC)] (Ru-1), immobilized within a HCP framework (HCP-Ru-1)	759 m^2^ g^−1^	Stainless steel autoclave, 150 °C, THF (5 mL), pentaethylenehexamine (PEHA, 5 mmol), H_2_ + CO_2_ (30:0 bar), and 48 h	CH_3_OH, 1150, and ~45% yield	10 cycles	[100]
PdAg nanoparticles supported on resorcinol-formaldehyde polymers (PdAg/amine-RF)	28.5 m^2^ g^−1^	Stainless autoclave, 40 °C, NaHCO_3_ in H_2_O (1.0 M, 15 mL), H_2_ + CO_2_ (1:1 MPa), and 24 h	HCOOH, 63, and not reported	3 cycles	[101]
Ru@POP-3	353.01 m^2^ g^−1^	Stainless steel reactor, 120 °C, aqueous Et_3_N solution (1.0 M, 5 mL), H_2_ + CO_2_ (1:1 MPa), and 2 h	HCOOH, 2049, and 0.15 M	5 cycles	[102]
Ru(III) complex coordinated-Troger’s base-derived microporous organic polymer (TB-MOP-Ru)	540 m^2^ g^−1^	Teflon-lined autoclave, 40 °C, Et_3_N (5 mL), PPh_3_ (1 mmol), H_2_ + CO_2_ (6:6 MPa), and 24 h	HCOOH, 2254, and 54.1 mmol	3 cycles	[103]
Ir@CTF	560 m^2^ g^−1^	Parr reactor, 90 °C, 0.5 M KHCO_3_, H_2_ + CO_2_ (1:1), and 2 h	HCOOH, 219, and not reported	4 cycles	[104]

Polymer-supported Ru, Ir, and Cu catalysts were investigated for selective CO_2_ hydrogenation under mild conditions in this study. For example, poly (4-vinylpyridine)-supported Ru–pincer complexes achieved full selectivity for formate at 70 °C and 40 bar (H_2_/CO_2_ = 1), with turnover numbers (TONs) exceeding 10,000, rivaling homogeneous benchmarks, while enabling simple catalyst recovery by filtration. The polymer support modulates the electronic density on the ‘Ru’ center through the trans-influence of the pyridyl nitrogen, and swelling in polar solvents ensures effective substrate access [100].

Amine-containing sorbent polymers have attracted considerable attention for catalyzing the hydrogenation of CO_2_ to produce formic acid or formate. In this context, Chen et al. reported the synthesis of Ru/p-dop-POM. The resulting material exhibited a relatively low surface area of 17.2 m^2^ g^−1^. The parent p-dop-POM exhibited moderate CO_2_ adsorption capacities of 9.0 mg g^−1^ at 273 K and 7.4 mg g^−1^ at 298 K, making it suitable for subsequent catalytic hydrogenation studies. The CO_2_ hydrogenation reactions were carried out in a Parr reactor at 120 °C, and formate was identified as the primary product of the reaction. Under these conditions, Ru/p-dop-POM achieved a formate concentration of 3.36 M and an impressive TON of 25,400. Because CO_2_ hydrogenation to form formic acid is an exothermic process, increasing the reaction temperature to 130 and 140 °C led to a decline in catalytic performance, likely due to the thermodynamic favorability of reverse reactions and reduced product stability at higher temperatures. For comparison, catalysts prepared by incorporating other metals, such as Ir, into the same cyclophosphazene polymer framework showed significantly lower activity, producing 1.46 M formate with a TON of 15,700. This highlights the superior performance of Ru-based heterogeneous systems, which is attributed to the synergistic interaction between electron-rich mononuclear Ru active sites and a nitrogen-rich polymer matrix. The nitrogen functionalities likely enhance CO_2_ adsorption and activation, thereby improving the catalytic efficiency. Overall, this study demonstrates that the strategic functionalization of polymer supports can significantly influence catalytic activity and product selectivity, providing valuable guidance for the design of advanced CO_2_ hydrogenation catalysts [97].

Ordered mesoporous polymers (OMPs) are an adaptable class of porous polymeric organic materials with large surface areas, precisely organized mesoporous structures, adjustable pore architectures, and chemically tunable skeletons. Their interlinked mesopore networks facilitate rapid mass transport and seamless access to active sites, making them prominent in heterogeneous catalysis and CO_2_ utilization technologies. The integration of functional groups or catalytically active centers within the porous polymer structure further improves their performance by enhancing chemical adsorption, active-site distribution, and catalyst selectivity [105,106].

Huidong et al. developed an efficient noble metal-based catalyst based on OMPs for CO_2_ hydrogenation to CH_3_OH as displayed in Figure 4. The corresponding amine-functionalized OMPs were synthesized via a soft-templating self-assembly approach, as shown in Figure 4a, using Pluronic F127 as a template and by partially substituting the resorcinol concentration with amine-containing phenol derivatives, such as 3-(3-aminopropyl)phenol or 3-aminophenol, yielding NOMP or SNOMP materials bearing primary amine functionalities. The ‘Pt’ nanoparticles were subsequently embedded onto the NOMP and SNOMP materials via wet impregnation, followed by reduction with NaBH_4_ to produce metallic ‘Pt’ components. The obtained catalysts were referred to as Pt/NOMP and Pt/SNOMP. The catalytic performance of Pt/SNOMP was investigated for the liquid-phase hydrogenation of CO_2_ to CH_3_OH using morpholine as a recyclable agent, and the resulting activity was very high compared to the performance of conventional catalysts (Pt/NOMP and Pd/SBA-NH_2_) in gas-phase fixed-bed reactors, as shown in Figure 4c. The conversion process involves a tandem two-step pathway, as shown in Figure 4b. In step 1, morpholine reacts with CO_2_ to produce N-formylmorpholine (NFM), achieving approximately 72% conversion after 24 h with an insignificant amount of CH_3_OH production. The selective formation of NFM was explained by the strong interaction of formate intermediates on the ‘Pt’ sites, which prevents further transformation or hydrogenation. Moreover, the maintained H_2_/CO_2_ ratio of 1:1 favored the formation of NFM. In step 2, NFM was hydrogenated under increased H_2_ pressure, yielding almost complete conversion within 12 h, and the CH_3_OH selectivity reached 99%, while only a minute amount of NFM was restored to morpholine and CO_2_. The CH_3_OH yield was ascribed to approximately 72% of the initial morpholine loading. Compared with previously studied catalysts, including Pt/NOMP and Pd/SBA-NH2, operating in gas-phase fixed-bed reactors, the Pt/SNOMP system in morpholine-assisted liquid-phase reactions showed considerably improved productivity. The conventional gas-phase fixed-bed reactor strategy is suppressed by the density of the surface amine groups accessible for CO_2_ capture. In contrast, the use of dissolved morpholine avoids this limitation and enables CH_3_OH yields of approximately 154.8 mmol g_cat_^−1^, representing an improvement of nearly three orders of magnitude. In addition, morpholine recovery surpassed 98%, and no appreciable liquid- or gas-phase by-products were observed. Moreover, the Pt/SNOMP catalyst also exhibited outstanding long-term stability up to 10 cycles without changes in the CH_3_OH yield and selectivity, as displayed in Figure 4d, confirming the durability of the catalyst and the robustness of the morpholine-mediated integrated CO_2_ capture and conversion approach.

The reaction mechanism is suggested in Figure 4e, which proceeds through the initial capture of CO_2_ by morpholine to form a carbamate intermediate, which subsequently undergoes hydrogenation to form NFM. This transition is considered the rate-determining step because of the high thermodynamic stability of the carbamate species and substantial energy barrier associated with C−O bond cleavage. Consequently, the apparent TOF during the first stage was relatively low. In the next step, the hydrogenation of NFM to CH_3_OH occurs much more rapidly, indicating that formamide reduction is kinetically favored once the intermediate is formed. Because both reaction stages rely on Pt-mediated H_2_ activation, intimate contact between ‘Pt’ nanoparticles and amine functionalities is essential for efficient tandem catalysis. This prerequisite also describes why the catalytic activity reaches stabilization with the 3 wt% ‘Pt’ loading, where sufficient ‘Pt’ reactive centers are already available to sustain the rapid turnover of the reaction intermediates [95].

HCPs are highly attractive porous materials because of their large surface areas and tunable pore architectures, both of which significantly contribute to their enhanced catalytic performance. These materials generally exhibit excellent stability during CO_2_ hydrogenation reactions and provide suitable porous environments for the immobilization of single-site molecular catalysts. Therefore, understanding the structural influence of molecular catalysts integrated within HCP frameworks is essential for improving their catalytic efficiency [77,100].

In this regard, Kumar et al. developed a Ru–N-heterocyclic carbene (Ru–NHC)-based homogeneous molecular catalyst, [RuHCl(CO)(PPh_3_)(bis-NHC)] (Ru-1), immobilized within an HCP framework (HCP-Ru-1), and systematically compared its CO_2_ hydrogenation activity with that of the parent homogeneous catalyst, as shown in Figure 5a [100]. The single-site heterogenized catalyst HCP-Ru-1 was synthesized through a hypercrosslinking polymerization strategy involving Ru-1 and triphenylmethane as arene monomers, while formaldehyde dimethyl acetal served as the crosslinking agent via a Friedel–Crafts reaction catalyzed by FeCl_3_ in 1,2-dichloroethane. The synthesized HCP-Ru-1 exhibited a high surface area of 759 m^2^ g^−1^ with pore sizes ranging from 0.5 to 0.8 nm and a CO_2_ adsorption capacity of 5.89 wt% at 273 K. The catalyst was subsequently evaluated for the hydrogenation of CO_2_ to CH_3_OH at 120 °C. Under identical reaction conditions, the parent Ru-1 catalyst displayed a TON of nearly 3520, whereas the heterogenized HCP-Ru-1 catalyst achieved a significantly higher TON of 15,750. This remarkable improvement clearly demonstrates the beneficial role of immobilization within the porous HCP matrix, which was proposed to protect the Ru–NHC catalytic centers from deactivation by H_2_O generated during the formylation step of the reaction. The recyclability and durability of HCP-Ru-1 were further investigated over ten consecutive cycles. The catalyst maintained excellent stability, exhibiting only a slight decrease in activity after 10-cycle reusability experiment. The minor decline in catalytic performance was mainly attributed to Ru leaching, with the Ru content decreasing from 0.41 to 0.33 wt%, as confirmed by ICP-OES.

As illustrated in Figure 5b, the catalytic cycle begins with the formation of an active Ru dihydride species through H_2_ activation in the presence of PEHA under basic conditions. Subsequently, CO_2_ inserts into the Ru–H bond to form a formate intermediate, which generates alkylammonium formate while regenerating the Ru species. The alkylammonium formate intermediate undergoes dehydration to produce formamide, which is then hydrogenated to form a hemiaminal intermediate. Finally, the hemiaminal decomposes into formaldehyde and amine, and subsequent hydrogenation converts the formaldehyde into methanol, continuously regenerating the active Ru catalyst throughout the process [100].

Owing to the unique structural and textural properties of HCPs, the same research group further developed hierarchical porous HCP-linked Ir-abnormal NHC (Ir-aNHC) catalysts for CO_2_ hydrogenation with H_2_ in H_2_O [96]. In contrast to their earlier study, formate was obtained as the major product, which is considered a promising candidate for direct formate fuel cell (DFFC) applications. Unlike previous systems that employed organic bases, this study utilized inorganic bases such as NaOH and KOH, offering significant practical advantages. As demonstrated by Prakash et al., the generated alkali metal formates (e.g., HCO_2_Na and HCO_2_K) can be directly employed in DFFCs without purification, enabling simultaneous electricity generation and regeneration of the corresponding hydroxides. The catalysts, Ir-HCP-(B) and Ir-HCP-(TPM), were synthesized through an FeCl_3_-catalyzed Friedel–Crafts alkylation method using B and TPM as copolymerizing agents, respectively. ICP-OES analysis revealed ‘Ir’ loadings of 0.420 wt% for Ir-HCP-(B) and 0.115 wt% for Ir-HCP-(TPM) catalysts. The BET surface areas of Ir-HCP-(TPM) and Ir-HCP-(B) were determined to be 1046 and 628 m^2^ g^−1^, respectively. Ir-HCP-(TPM) exhibited hierarchical pore sizes of 1.5 and 3.8 nm, whereas Ir-HCP-(B) exhibited a single pore size of 1.5 nm. Owing to its larger surface area and hierarchical porosity, Ir-HCP-(TPM) demonstrated superior catalytic performance compared to Ir-HCP-(B). Under optimized conditions in the presence of KOH, Ir-HCP-(TPM) achieved an impressive TON of 50,816, producing 6098 µmol of formate at 80 °C in a single reaction cycle over 4 h. In comparison, Ir-HCP-(B) delivered a TON of 9733 with 1168 µmol of formate in 1 h. Notably, the catalytic activity of Ir-HCP-(TPM) surpassed that of the corresponding homogeneous Ir-aNHC catalyst, which exhibited a TON of only 2666 with 320 µmol of formate produced in 1 h. Furthermore, the Ir-HCP-(TPM) catalyst displayed excellent long-term stability after ten cycles. Only a slight activity loss of approximately 1.6% per cycle was observed, highlighting its excellent recyclability and durability. The robustness of the catalyst was further confirmed by hot filtration tests, which supported the heterogeneous nature and stability of the catalyst during the reaction [96].

### 4.4. Integrated CO_2_ Capture and Catalytic Conversion: Dual-Functional Polymer Systems

Dual-functional polymers capable of capturing and converting CO_2_ within the same material represent an elegant class of materials. These systems typically combine a CO_2_-philic adsorption component (amine, ionic liquid, or basic nitrogen site) with a catalytically active component (Lewis acid, metal complex, or organocatalytic unit) in a single framework. The most successful examples are amine–Lewis acid cooperative POPs, in which the amine captures CO_2_ as a carbamate intermediate, which is then activated by the neighboring Lewis acid for reaction with an epoxide to form an oxazolidinone or a CC. This tandem adsorption-conversion mechanism is feasible at significantly lower CO_2_ pressures than purely catalytic systems that do not incorporate a capture function.

**Table 6:** Representative functional polymer catalysts for integrated CO_2_ capture and conversion: Performance metrics and recyclability.

Polymer Catalyst/Sorbent	Surface Area	Application	Reaction Conditions	Performance Metric (TON or TOF (h^−1^), and Yield or Conversion)	Recyclability (Cycles)	Reference
JNM-Au-1 (JNM = Jinan Materials)	351.01 m^2^ g^−1^	Carboxylation of alkynes	50 °C, DMF (1 mL), Cs_2_CO_3_ (1.1 equiv.), 1 atm of CO_2_, and 2 h	80.4 h^−1^ and up to 92% yield	5 cycles	[107]
[Ag(tpy)]_5_[Nb_10_O_28_][Ag(H_2_O)]·5H_2_O (tpy = 2,2′:6′,2″-terpyridine) coordination polymer	Not reported	Carboxylative cyclization of propargyl alcohols	25 °C, Ph_3_P (0.06 mmol), 1 MPa of CO_2_, and 10 h	5144 and up to 99% yield	5 cycles	[108]
Polymer-supported 1,8-diazabicyclo [5.4.0] undec-7-ene (PS-DBU)	Not reported	Carbamate synthesis	Room temperature, CH_3_CN (7 mL), CH_3_CH_2_I (2.4 eq.), CO_2_ (balloon), and 18 h	Not reported and up to 95% yield	8 cycles	[109]
Ru@PGS (PGS = polymer-grafted SiO_2_)	84 m^2^ g^−1^	Hydrogenation of furfural acetone	In-house built continuous-flow reactor, 80 °C, 1-butanol (0.5 mL), H_2_ + CO_2_ (15:15 bar), and 12 h	Not reported and >99% conversion	6 cycles	[110]
[Eu(tctb)(H_2_O)]⋅guest molecules (SION-105)	216 m^2^ g^−1^	Cyclization of o-phenylenediamines	Teflon lined autoclave, 120 °C, CH_3_CN (2 mL) and Ph_2_SiH_2_ (0.8 mmol), 1 MPa of CO_2_, and 24 h	Not reported and up to 99% conversion	5 cycles	[111]
Ru-supported 2,6-di(9*H*-carbazol-9-yl)pyridine (CarPy) CMPs (CarPy-CMP@Ru)	735 m^2^ g^−1^	Formylation of amines	Teflon lined autoclave, 130 °C, CH_3_OH (3 mL), H_2_ + CO_2_ (4:4 MPa), and 24 h	Not reported and up to 97% conversion	5 cycles	[112]
Ag-supported thiadiazole-based conjugated organic polymer (Ag@BT-COP)	187.8 m^2^ g^−1^	Carboxylative cyclization of propargylic amines to 2-oxazolidinones	Reaction flask, 60 °C, CH_3_CN (3 mL) and DBU (0.5 equiv.), CO_2_ (balloon), and 14 h	Up to 605.3 and up to >99% conversion	5 cycles	[113]
Cu-based N-heterocyclic carbene porous polymer catalyst (Cu@NHC-1)	341.4 m^2^ g^−1^	Carboxylative cyclization of propargylic amines to 2-oxazolidinones	Continuous flow device, 50 °C, CH_3_CN (4 mL), mixture of 1 bar CO_2_/dry air (30 vol% CO_2_), and 7.5 h	Up to 74 and up to 96% yield	5 cycles	[114]
Ag-supported N-rich POP (Ag@NPOP-1)	481 m^2^ g^−1^ (Of NPOP-1)	Carboxylation of alkynes	60 °C, DMSO (1 mL) and CS_2_CO_3_ (0.6 mmol), CO_2_ (balloon), and 12 h	Up to 90.9 h^−1^ and up to 94.2% yield	5 cycles	[115]
Poly(*N,N*′-diallyl-*N,N′*-dimethylammonium carboxylate) (PDDC)	164.2 m^2^ g^−1^	*N*-methylation of amines	Glass stopcock equipped reaction tube, 60 °C, CH_3_CN (2 mL) and PhSiH_3_ (1.5 mmol), 1 bar of CO_2_, and 10 h	Not reported and up to 93% conversion	3 cycles	[116]
CuCl_2_@Poly-GLY(1-Vim)_3_(OMs)_3_	86 m^2^ g^−1^	Carboxylation of alkynes	40 °C, Cs_2_CO_3_ (0.62 g), n-BuI (0.89 g), 4 MPa of CO_2_, and 12 h	Not reported and up to 96% yield	4 cycles	[117]

Before discussing specific systems, it is important to distinguish between genuinely integrated tandem catalysis, where CO_2_ capture and conversion occur concurrently at the same material, often at similar temperatures without intermediate product isolation, and sequential two-step processes, where capture and conversion are conducted under different conditions. True dual-functionality requires (i) CO_2_ capture capacity under the conversion conditions (temperature, pressure, solvent) and (ii) catalytic conversion activity that demonstrably benefits from the in-situ captured CO_2_ rather than from separately supplied CO_2_. The Cu@NHC-1 system [98] satisfies both criteria: it captures CO_2_ from 30 vol% CO_2_/air feeds at 50 °C and converts it within the same reactor without CO_2_ preconcentration. In contrast, several systems in Table 5, such as PS-DBU [93] and JNM-Au-1 [91], use CO_2_ as a reagent under pressurized conditions without a distinct capture step and are better described as CO_2_-utilizing catalysts rather than integrated CCU systems. Isotopic labelling studies using ^13^CO_2_ would be definitive in confirming true tandem operation, and this represents an important gap in the current literature.”

A key advantage of integrated carbon capture and conversion is its ability to integrate both CO_2_ capture and catalytic conversion within a single material system, making the process highly attractive from both economic and practical perspectives. Functional polymer catalysts are particularly promising for this purpose because their tunable surface chemistry and multifunctional active sites enable them to simultaneously adsorb CO_2_ and catalyze its transformation into value-added products [5,42,56]. In this context, Bordet et al. [110] developed an advanced catalytic system integrated with a continuous-flow reactor for the selective hydrogenation of biomass-derived furfural acetone under H_2_ and H_2_/CO_2_ atmospheres. The system enabled controllable product selectivity depending on the reaction atmosphere. To achieve this switchable selectivity, the authors designed a highly active and stable catalyst composed of Ru nanoparticles immobilized on tertiary amine-functionalized polymer-grafted SiO_2_, denoted Ru@PGS (Figure 6a). The catalyst had a surface area of 84 m^2^ g^−1^. The polymer-grafted silica support acted as a CO_2_-responsive material that played a key role in tuning the reaction pathway in different gaseous environments. In a CO_2_/H_2_ mixture, the reaction selectively produced 4-(tetrahydro-2-furyl)butan-2-one, whereas direct hydrogenation under pure H_2_ predominantly yielded 4-(tetrahydro-2-furyl)butan-2-ol, as shown in Figure 6c,d. In both cases, the conversion exceeded 99% at 100 °C, and the product distribution was governed by the gas composition. In the presence of a CO_2_/H_2_ atmosphere, alkylammonium formate species were formed on the Ru@PGS surface, altering the catalytic pathway and directing the formation of the ketone product. Importantly, the continuous-flow reactor system could operate in two reversible and switchable modes (Figure 6b–d). By simply alternating the gas feed between pure H_2_ and an H_2_/CO_2_ mixture, the product selectivity could be reversibly controlled during continuous operation without loss of catalytic performance. This study highlights the potential of CO_2_-responsive functional polymer surfaces as catalyst supports for metal nanoparticles and provides valuable insights into the development of adaptive catalytic systems for sustainable energy conversion and industrial CO_2_ utilization technologies [110].

In the pharmaceutical industry, oxazolidinones are important intermediates in organic synthesis and antimicrobial drug production processes. Their synthesis via CO_2_ fixation using propargylamines is particularly attractive because it provides economic value and environmental benefits. However, industrial waste gases contain CO_2_ at low concentrations, and its direct on-site conversion into value-added products from mixed gas streams remains a significant challenge [118,119]. To address this, Rao et al. [114] developed a Cu@NHC-1 porous polymer for onsite conversion of low-concentration CO_2_ using a model lime-kiln waste-gas mixture. A continuous-flow system was designed to supply a steady stream of dilute CO_2_ (approximately 30 vol. %) to the reaction system. Owing to the cooperative effects of the Cu active centers, nitrogen functional groups, and porous framework, the Cu@NHC-1 catalyst achieved excellent performance, yielding oxazolidinones with yields of up to 96%. The Cu@NHC-1 catalyst exhibited a surface area of 341.4 m^2^ g^−1^ with a predominant pore diameter of less than 5 nm. Its CO_2_ adsorption capacity was measured to be 13.39 cm^3^ g^−1^ at 298 K. Thermogravimetric analysis (TGA) confirmed that the material was thermally stable, with no significant weight loss observed up to 400 °C. As illustrated in Figure 6e, CO_2_ was initially captured and enriched within the porous framework of Cu@NHC-1, where it was activated by the nitrogen-containing functional groups. Simultaneously, propargylamine molecules were confined within the porous structure and coordinated to the active ‘Cu’ centers. The copper sites further interact with the C≡C bond of propargylamine, forming an activated π-complex intermediate (3), which facilitates the nucleophilic attack and drives the reaction forward [114].

Similar to oxazolidinones, carbamates are highly important nitrogen-containing compounds with wide applications in the chemical, pharmaceutical, and agrochemical industries. They also serve as efficient amine-protecting groups and key intermediates in peptide synthesis and other organic transformations [120]. Conventionally, their synthesis often requires strong organic bases such as DBU and 1,1,3,3-tetramethylguanidine (TMG); however, these systems suffer from limitations, including poor catalyst recovery, high cost, and the need for elevated temperatures and CO_2_ pressures. To address these challenges, Antonio et al. reported a reusable PS-DBU system for the synthesis of CO_2_-inserted carbamates with CO_2_ insertion. In this approach, DBU is anchored to a polymer support and employed as a robust heterogeneous catalyst for the formation of alkyl carbamates. The developed catalyst can be reused for multiple cycles without significant loss of activity, eliminating the need for conventional purification steps. The reaction proceeds efficiently in a one-pot process at room temperature under low CO_2_ pressure, delivering carbamates with yields of up to 95%. As illustrated in Figure 6f and based on previous NMR studies by Liotta et al., the proposed mechanism proceeds through two possible pathways. In the main pathway (a), CO_2_ is initially activated by the amine, followed by deprotonation by the strong, hindered DBU base to form a carbamic carboxylate intermediate, which undergoes alkylation to yield the corresponding carbamate. Alternatively, the carbamic carboxylate intermediate may be generated via the competing pathway (b), which can lead to product formation. Overall, this study highlights the potential of polymer-supported DBU in carbamate synthesis, offering high efficiency, operational simplicity, effective CO_2_ utilization, and the added advantage of catalyst recyclability [109].

Furthermore, efforts have been made to design catalytic polymer membranes for integrated CO_2_ capture and conversion. Hui et al. developed a polymer membrane based on quaternized poly(4-vinylpyridine) (P4VP) for selective CO_2_ separation and CO_2_ cycloaddition with epoxides to form the respective CCs in a continuous flow membrane reactor. The membrane must demonstrate selective and effective CO_2_ separation, high catalytic activity, and long-term operational stability of the membrane. According to previous reports, the P4VP catalytic membrane displayed good CO_2_ separation and high catalytic conversion of epoxides with CO_2_ under mild conditions. However, its stability is poor because of its high solubility in ECH. To overcome this problem, in this study, a quaternized derivative, P4VP-C2-HCO_3_, was synthesized through ethylation to form P4VP-C2-Br and subsequent bicarbonate ion exchange. The addition of bicarbonate ions to the membrane remarkably improved the separation performance (109 ± 6) at 57 °C when compared to the P4VP-C2-Br catalytic membrane, indicating outstanding CO_2_ discrimination even for dilute CO_2_ streams. Catalytic testing of P4VP-C2-HCO_3_ revealed the highest CC production rate (10.3 ± 0.6 mmol h^−1^ active site^−1^) compared to those of the P4VP-C2-Br and pristine P4VP catalytic membranes. These results demonstrate that quaternization and anion exchange can modify the catalytic environment without altering the fundamental active sites associated with the pyridine groups required for the CO_2_ cycloaddition with epoxides. Therefore, the catalytic performance of P4VP-based materials can be tuned through targeted chemical modifications. Furthermore, the P4VP-C2-HCO_3_ catalytic membrane exhibited significant long-term stability and retained its activity during continuous operation for up to 48 h, despite some gradual deactivation during the first 30 h of operation before reaching a stable value. The reason for this decline is evidenced by ATR-FTIR spectroscopic analysis, which revealed the accumulation of by-products, such as glycidol and 3-chloro-1,2-propanediol, on the membrane surface. These strongly adsorbed species favourably contribute to partial catalyst deactivation, although the membrane retained steady activity during extended operation, highlighting its potential for integrated CO_2_ capture and utilization processes [6].

For efficient CO_2_ conversion, future advances in operando spectroscopy combined with DFT investigations are expected to provide deeper insights into catalyst-specific rate-determining steps and activation barriers, enabling the strategic design of next-generation polymer catalysts.

## 5. Catalyst Stability, Recyclability, and Sustainability Metrics

The practical value of any functional polymer catalyst for CO_2_ capture or conversion depends critically on its ability to be recovered and reused without a significant loss of performance. The catalysts and sorbents reviewed in Section 4 span a wide range of deactivation behaviors and recyclability profiles, as summarized in Table 6. This section draws directly on these systems to identify the dominant deactivation mechanisms, evaluate the regeneration strategies reported in the cited studies, and assess sustainability using green chemistry metrics.

### 5.1. Catalyst Lifetime and Reuse: Evidence from Reviewed Systems

The CO_2_ capture sorbents reviewed in Section 4.1 demonstrated the most consistent recyclability. The O-doped HAT-CTF reported by Zhu et al. [69] retained >98% of its CO_2_ uptake capacity over five adsorption–desorption cycles, which was attributed to the thermal stability of the triazine framework and the absence of irreversible chemical bonding between the sorbent and CO_2_ molecules. PEI@PGD-H maintained >97% capacity over 20 cycles when regenerated at 90–100 °C [68], and the ionic lignin polymer of Ghorai and Chung [74] retained >95% performance over four cycles with regeneration at only 60 °C, owing to the reversible bicarbonate-forming chemisorption mechanism.

For CO_2_ cycloaddition catalysts (Section 4.2), the recyclability data were consistently strong across the reviewed systems. The HCPs-HQ catalyst [80] and the Zn-HUT4 ionic POP [81] both maintained >95% yield and conversion over 5–6 cycles, with no evidence of imidazolium or Zn leaching by post-cycle characterization (BET, FT-IR, and ICP-OES). The CAP-DAP covalent aromatic polymer [84] retained >95% conversion over six cycles, and the MMFR and MMFR250 melamine–formaldehyde resins [83] operated continuously for over 100 h in flow without a loss of selectivity. The PIL-Br_1.0_@Zn-CTM nanocomposite [89] maintained excellent performance over seven cycles under dilute 15% CO_2_ conditions, confirming the robustness of the poly(ionic liquid) coating on the Zn-CTM support surface.

For CO_2_ hydrogenation catalysts (Section 4.3), a gradual but well-characterized activity decline was observed across cycles, which was attributable to trace metal leaching from the polymer matrix. HCP-Ru-1 lost approximately 1.1% of its activity per cycle over ten runs, corresponding to a decrease in Ru content from 0.41 to 0.33 wt%, as confirmed by ICP-OES measurements [100]. Similarly, Ir-HCP-(TPM) showed a 1.6% activity loss per cycle over ten runs but achieved a remarkable cumulative TON of 285,400 for CO_2_-to-formate production [96]. Hot filtration tests confirmed that the catalysis remained fully heterogeneous, establishing that minor leaching did not generate a competing homogeneous catalytic phase.

### 5.2. Deactivation Mechanisms Specific to the Reviewed Catalysts

The analysis of the reviewed systems identified four dominant deactivation mechanisms: (i) Metal leaching from polymer–metal hybrid catalysts: observed in both HCP-Ru-1 [100] and Ir-HCP-(TPM) [96] during CO_2_ hydrogenation, the ‘Ru’ and ‘Ir’ centres gradually dissociate from their NHC chelating ligands under high H_2_ pressure and basic aqueous conditions. Choudhury et al. demonstrated that encapsulation within a dense HCP matrix significantly retards this process by sterically shielding the metal–NHC bond from competing ligand exchange. (ii) Amine oxidation and urea formation in CO_2_ capture sorbents: primary amine groups in PEI@PGD-H are susceptible to oxidation in the presence of O_2_ at temperatures above 120 °C. The studied systems avoid this by restricting regeneration to below 100 °C [68]. In dual-functional POPs [121], adjacent amine groups can form stable urea linkages at elevated temperatures, irreversibly reducing the CO_2_ capture capacity. Furthermore, amine oxidation and urea formation can be mitigated by using secondary amines instead of primary amines or by applying steam during the regeneration process [122,123].

In addition, degradation associated with steam exposure can be reduced by employing porous supports with superior hydrothermal stability. (iii) Substrate fouling in cycloaddition catalysts: MMFR systems [83] showed minor deactivation under extended continuous-flow operation, which was attributed to the adsorption of higher-molecular-weight epoxide oligomers within the mesoporous channels. A brief N_2_ purge at 150 °C fully restored the catalytic activity. (iv) Amine leaching remains a major limitation during the cycling operation of impregnated amine-based CO_2_ sorbents. Hicks et al. demonstrated that TEPA-impregnated uncalcined SBA-15 (SBA-UCTEPA) experienced a progressive decrease in CO_2_ uptake after the first cycle owing to the loss of physically adsorbed TEPA during repeated adsorption–desorption operations [124]. To mitigate this challenge, the covalent grafting of amine species on SiO_2_ supports is an excellent strategy for developing robust CO_2_ sorbents, as it combines reversible CO_2_ capture with enhanced resistance to amine loss and improved long-term operational stability [125]. (v) Metal leaching is another major challenge in polymer–metal hybrids for catalytic CO_2_ conversion that affects the efficiency and reusability of the catalyst as shown in Table 7. The HCP-Ru-1 catalyst displayed an activity loss of ~1.1% per cycle over ten recycling cycles, which correlated with a decrease in Ru content from 0.41 to 0.33 wt% according to ICP-OES measurements [100]. To address the challenge of metal leaching, the integration of ionic liquid (IL) functionalities into metalloporphyrin-based porous organic polymers (MPOPs) to yield IL-MPOPs has emerged as an effective strategy. The weak coordination nature of the cationic IL groups minimizes metal loss during catalysis, thereby enhancing structural stability and recyclability. In particular, imidazolium moieties function as H_2_-bond donors and nucleophilic promoters, facilitating substrate activation and lowering the energy barriers. Furthermore, free radical polymerization offers a sustainable, metal-free route for the synthesis of IL-MPOPs, providing high monomer versatility, operational simplicity, and excellent atom economy. Consequently, these architectures exhibit remarkable structural robustness and prolonged catalytic performance, highlighting their potential for sustainable carbon capture and utilization applications [126,127]. (vi) Competitive water adsorption in sorbents: Although the ionic lignin polymer [74] showed excellent CO_2_ selectivity over water in direct air capture experiments, the PEI-based sorbents are known to be sensitive to high humidity. In Section 4.1, MCF@G-PEI [66] maintained 2.78 mmol g^−1^ at 50% RH, demonstrating that co-grafting strategies can mitigate water competition.

**Table 7:** The stability and recyclability of the functional polymer catalyst and sorbent systems are reviewed in Section 4, including CO_2_ conversion applications, cycles reported, performance retention, and key deactivation mechanisms.

Catalyst/Sorbent System	CO_2_ Conversion Application	Cycles/Time	Activity/Capacity Retention (%)	Key Deactivation Mechanism and Mitigation Strategy	Reference
O-doped HAT-CTFs-450/600	CO_2_ capture (post-combustion)	5 cycles	>98	Mild physisorption, thermal regeneration at 70–100 °C fully restores capacity, and no structural degradation by PXRD	[69]
PEI@PGD-H	CO_2_ capture	20 cycles	>97	Amine oxidation at >120 °C and mitigated by regeneration below 100 °C under N_2_	[68]
Quaternary ammonium-modified ionic lignin polymer	CO_2_ capture and release	4 cycles	>95	Mild chemisorption via bicarbonate, CO_2_ released at 60 °C, and stable quaternary ammonium linkage	[74]
HCPs-HQ	CO_2_ cycloaddition to epoxides	6 cycles	>95	No leaching detected, imidazolium units stable, and pore structure confirmed by BET post-cycle	[80]
Zn-HUT4	CO_2_ cycloaddition to epoxides	5 cycles	>99 (conv.)	Stable Zn coordination to N/O framework, structural integrity by XRD, and minor loss attributed to substrate adsorption	[81]
CAP-DAP	CO_2_ cycloaddition to epichlorohydrin	6 cycles	>95	H-bond donor/acceptor groups stable, no leaching, and mild regeneration by washing with ethanol/water	[84]
MMFR/MMFR250	Continuous-flow CO_2_ cycloaddition	>100 h (flow)	>99 (sel.)	Basicity stable under continuous operation, minor deactivation by heavy epoxide oligomers, and regenerated by N_2_ purge	[83]
PIL-Br_1.0_@Zn-CTM	CO_2_ cycloaddition	7 cycles	>95	Robust C–N framework, Br^−^ nucleophile stable, and no significant Zn leaching confirmed by ICP-OES	[89]
HCP-Ru-1	CO_2_ hydrogenation to methanol	10 cycles	~89 (TON loss 1.1%/cycle)	Minor Ru leaching (0.41 → 0.33 wt% by ICP-OES) and HCP pore matrix protects NHC from H_2_O deactivation	[100]
Ir-HCP-(TPM)	CO_2_ hydrogenation to formate	10 cycles	~84 (loss 1.6%/cycle)	Hot-filtration test supported heterogeneous catalysis, Ir stable in KOH, and cumulative TON 285,400	[96]
Ru@PGS	CO_2_-responsive switchable hydrogenation to furfural acetone	6 cycles	>99 (conv.)	Tertiary amine support stable, selectivity fully switchable H_2_ vs. CO_2_/H_2_, and no activity loss over 150 h	[110]
Cu@NHC-1	Carboxylative cyclization to oxazolidinone	5 cycles	>90	Stable to 400 °C by TGA, N-rich framework retains CO_2_ affinity, and Cu chelation by NHC prevents leaching	[114]

### 5.3. Regeneration Strategies Demonstrated in Section 4 Systems

The regeneration methods employed in the reviewed systems can be classified into four approaches. Temperature swing adsorption (TSA) at 90–120 °C under inert gas is the most common strategy for CO_2_ capture sorbents, used for PEI@PGD-H [68], O-doped HAT-CTFs-450/600 [69], and amine-modified HCPs [27]. The ionic lignin polymer [74] demonstrates a milder alternative: CO_2_ release at 60 °C by gentle heating, enabled by the reversible bicarbonate-forming mechanism of the quaternary ammonium groups. For flow-mode cycloaddition catalysts (MMFR series [83]), in situ regeneration by periodic N_2_ purging at elevated temperatures within a continuous-flow reactor was demonstrated to be practical and energy efficient. For polymer–metal hydrogenation catalysts, re-immersion in a fresh metal precursor solution followed by mild reduction can restore activity; this strategy is feasible for HCP-Ru-1, given the synthetic accessibility of the Ru-NHC precursor [100]. For the Ru@PGS system [110], CO_2_-responsive regeneration is built into the catalytic cycle: alternating the gas feed between CO_2_/H_2_ and pure H_2_ reversibly switches both the product selectivity and sorbent saturation state without interrupting the flow, representing a conceptually advanced approach to integrated catalyst regeneration.

### 5.4. Green Metrics: Assessment Across Reviewed Systems

The reviewed systems collectively illustrate the three sustainability dimensions. First, atom economy and E-factor: CO_2_ cycloaddition to epoxides is inherently 100% atom-economical, and the polymer catalysts reviewed (Zn-HUT4, HCPs-HQ, MMFR, and CAP-DAP) operate under solvent-free or low-solvent conditions, thereby minimizing waste. The MMFR system [83] is particularly notable for its use under continuous-flow conditions without added co-solvents, resulting in a process E-factor below 0.3. Second, energy efficiency: the Ir-HCP-(TPM) system [96] uses KOH as the sole base additive and operates at 80 °C, well below the 120–160 °C required by most inorganic heterogeneous CO_2_ hydrogenation catalysts, which significantly reduces the thermal energy input. Third, catalyst reusability is the primary sustainability lever. The cumulative TON of 285,400 achieved by Ir-HCP-(TPM) [96] and the 20-cycle operation of PEI@PGD-H [68] with minimal material consumption demonstrate that polymer catalysts can deliver high productivity per gram of material synthesized, which is a key metric for lifecycle assessment. The biomass-derived ionic lignin polymer [74] adds a further sustainability dimension by sourcing the catalyst from industrial lignin waste, contributing to the principles of a circular carbon economy.

## 6. Comparison with Other Catalyst Platforms for CO_2_ Applications

A direct quantitative comparison of the polymer catalysts reviewed in Section 4 with MOF- and COF-based CO_2_ cycloaddition catalysts is not straightforward because the primary literature reports these systems under substantially different testing conditions, including different epoxide substrates, temperatures (40–220 °C), CO_2_ pressures (0.1–4.0 MPa), co-catalyst identities and loadings, and solvent environments. Therefore, the polymer catalysts presented in this review are compared with other catalyst platforms qualitatively. Table 8 illustrates the differences between polymer catalysts and other systems in terms of the properties and cost involved in the synthesis process.

**Table 8:** The qualitative comparison of polymer functional catalysts with MOFs and COFs.

Dimension	Functional Polymer Catalysts	MOFs	COFs
Synthesis cost and complexity	Low–moderate (USD 5–80 kg^−1^); aqueous or solvent-free routes available; no precious-metal catalyst required for HCPs and resins	High (USD 150–600 kg^−1^); DMF or hydrothermal synthesis; toxic precursors (Cr, Zr) common	High (USD 100–450 kg^−1^); slow imine condensation (up to 72 h); inert atmosphere required
Chemical and hydrothermal stability	CTF and HCP backbones stable under acid, base, and humid conditions; confirmed by post-cycling BET and PXRD	Zr-MOFs (UiO-66 series) stable pH 2–10; most MOFs degrade in moisture or steam	Imine-linked COFs susceptible to hydrolysis; β-ketoenamine COFs more robust
Compatibility with dilute CO_2_ streams	PIL-Br@Zn-CTM [80] active at 15% CO_2_ without pre-concentration; Cu@NHC-1 [98] uses 30% CO_2_ from waste gas	Most MOF catalysts require concentrated or pressurized CO_2_; activity drops sharply at low pCO_2_	Limited data under dilute CO_2_; most studies use 1 bar pure CO_2_
Recyclability (reported cycles)	5–22 cycles with >94–99% retention across reviewed systems	Typically 3–6 cycles; leaching and framework degradation limit longer runs	Typically 3–5 cycles; hydrolytic instability limits recyclability in humid conditions
Scalability of synthesis	Kilogram-scale HCPs and resins commercially available (Amberlyst, Dowex); hydrothermal POP synthesis scalable	Gram-to-hundred gram scale in most reports; scale-up hampered by DMF cost and Zr/Cr precursor handling	Milligram-to-gram scale in most reports; slow kinetics and batch variability limit scale-up

Furthermore, despite impressive lab-scale performance, the translation of functional polymer catalysts to industrial deployment requires addressing their processability and engineering challenges. POP-based sorbents and catalysts are typically obtained as fine powders unsuitable for direct packing into fixed-bed adsorbers owing to their unacceptable pressure drop. Pelletization using binders (polyvinyl alcohol and bentonite) or spray-drying can reduce the BET surface area by 10–30% through pore blockage. The mechanical stability of TSA systems under repeated compression–decompression cycles must be verified by attrition testing (ASTM D4058). Solvent compatibility is a further concern; crosslinked polymer catalysts that swell in DMF or DMSO can experience pore collapse upon drying, reducing the accessible surface area for subsequent cycles. The MMFR system [74] demonstrates that hydrothermal synthesis routes can deliver mechanically robust mesoporous resins directly suitable for continuous-flow operation without pelletization, which is a promising design strategy for scalable manufacturing.

## 7. Conclusions

This review presents a focused and evidence-based analysis of functional polymer catalysts for CO_2_ capture and conversion, covering four major material classes and four catalytic application areas: CO_2_ adsorption, CO_2_ cycloaddition to epoxides, CO_2_ hydrogenation, and CO_2_ integrated dual-functional conversion. The central finding is that the tunability of polymer architecture−backbone chemistry, functional group type and density, pore structure, and microenvironmental effects− enables performance that matches or exceeds inorganic and crystalline porous materials while offering unique advantages in synthesis accessibility, biomass derivability, stimuli-responsiveness, and compatibility with dilute CO_2_ streams.

Among the CO_2_ capture sorbents reviewed, O-doped HAT-CTF and PEI@PGD-H stand out for their exceptional uptake capacities (4.8 and 4.3 mmol g^−1^, respectively) and outstanding cyclability (>98% and >97% retention, respectively). The biomass-derived ionic lignin polymer demonstrates that excellent CO_2_ capture and release (at just 60 °C) is achievable from waste feedstocks, pointing toward a circular approach to CO_2_ management. Among cycloaddition catalysts, Zn-HUT4 ionic POP, HCPs-HQ, and MMFR melamine–formaldehyde resins collectively illustrate that high TOFs (up to >2000 h^−1^), near-quantitative conversions, and long operational lifetimes are simultaneously achievable in metal-containing and metal-free polymer frameworks, including under continuous-flow conditions relevant to industrial deployment. The PIL-Br_1.0_@Zn-CTM system pushes the frontier further by achieving catalytic cycloaddition under realistic 15% CO_2_ flue gas conditions without CO_2_ pressurization.

For CO_2_ hydrogenation, the Ir-HCP-(TPM) catalyst demonstrated that metal–NHC complexes immobilized within HCP matrices delivered a cumulative TON of 285,400, which compares favorably with the best homogeneous benchmarks while offering straightforward solid-phase recovery. The Ru@PGS system represents a conceptually distinct achievement: a CO_2_-responsive polymer support that reversibly controls product selectivity in a continuous-flow reactor by simply altering the gas-phase CO_2_/H_2_ ratio, exemplifying the programmable catalysis that is unique to stimuli-responsive polymer platforms.

In the dual-functional and integrated CO_2_ utilization category, Cu@NHC-1 and the PS-DBU system demonstrated that polymer catalysts can utilize low-concentration CO_2_ from industrial waste gases under mild atmospheric-pressure conditions, converting it to pharmaceutically relevant oxazolidinones and carbamates. These results challenge the assumption that CO_2_ valorization requires pressurized pure CO_2_ feeds.

The key design principles identified across these systems—cooperative Lewis acid–Lewis base site proximity for CO_2_ cycloaddition activation, CO_2_-philic microporous environments for concentrating dilute CO_2_ at active sites, N-rich and O-doped backbones for elevated isosteric heats of adsorption, hydrophobic coatings for humidity tolerance, and the HCP matrix as a protective scaffold for single-site metal catalysts-provide a practical toolkit for designing the next generation of polymer CO_2_ materials. The challenges that remain, as discussed in Section 7, of closing structure–activity gaps, performing operando characterization under realistic flue gas or direct air capture conditions, and scale-up challenges are substantial but tractable, given the rapid pace of progress in polymer synthesis and computational materials science.

Functional polymer catalysts are uniquely positioned to contribute to industrial CO_2_ mitigation as they combine the chemical versatility required for the full spectrum of CO_2_ capture and conversion applications, recyclability, mild-condition regeneration required for viability, and synthetic accessibility from low-cost and biomass-derived monomers, which enables production at the scale demanded by climate action. Realizing this potential will require sustained interdisciplinary collaboration between polymer chemists, chemical engineers, computational scientists, and industrial process developers, a challenge that the systems reviewed here demonstrate the field is increasingly well-equipped to meet.

## Figures and Tables

**Scheme 1:**
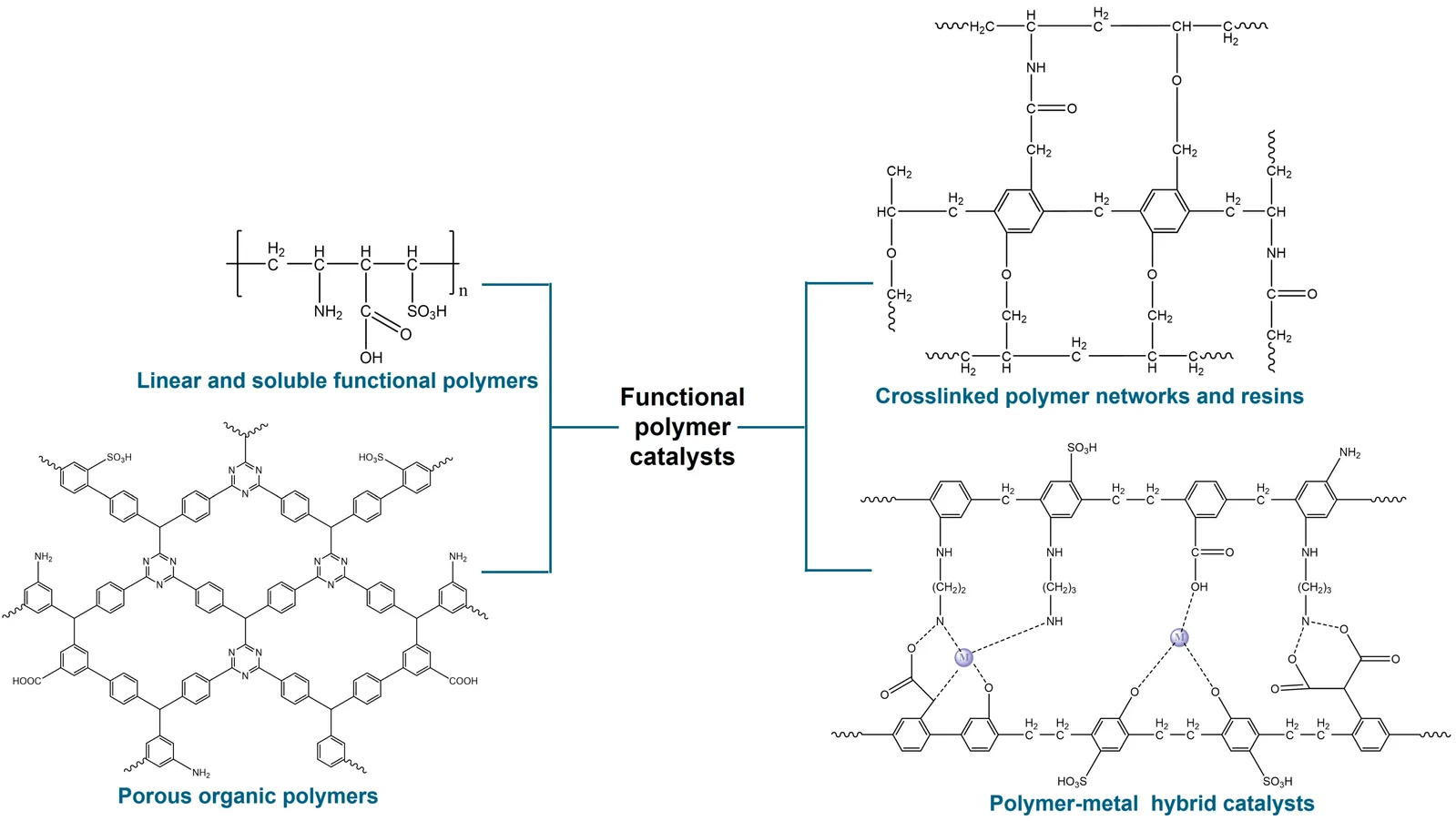
Common structural representations of different classes of functional polymer catalysts.

**Figure 1:**
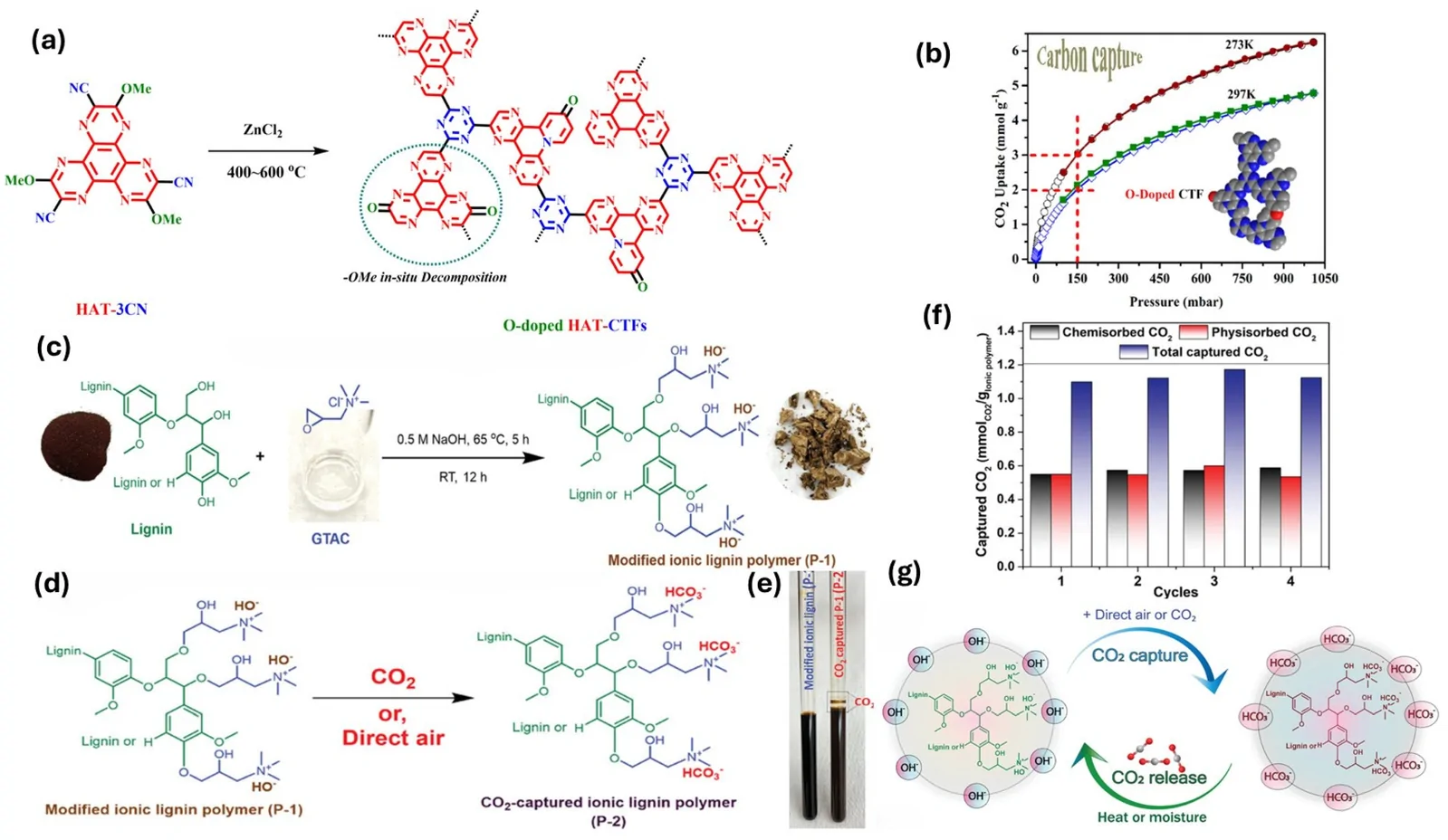
Functional polymer catalysts for CO_2_ capture: (**a**) synthesis route of O-doped HAT-CTFs, (**b**) CO_2_ uptake of O-doped HAT-CTFs at 273 and 297 K under 1 bar, reproduced with permission from [69]. Copyright 2016 ACS. (**c**) Synthesis scheme of the modified ionic lignin polymer (P-1) from GTAC and lignin under basic conditions, with associated physical changes. (**d**) CO_2_ capture behaviour of ionic lignin polymer (P-2) from concentrated CO_2_ sources and direct air capture. (**e**) NMR tube images of the polymer before and after CO_2_ adsorption in D_2_O. (**f**) CO_2_ capture efficiency of the regenerated ionic lignin polymer after four consecutive capture–release cycles. (**g**) Schematic representation of the recyclability of the ionic lignin polymer through repeated CO_2_ capture–release cycles. Adapted from [74]. Copyright 2024 Wiley-VCH GmbH.

**Figure 2:**
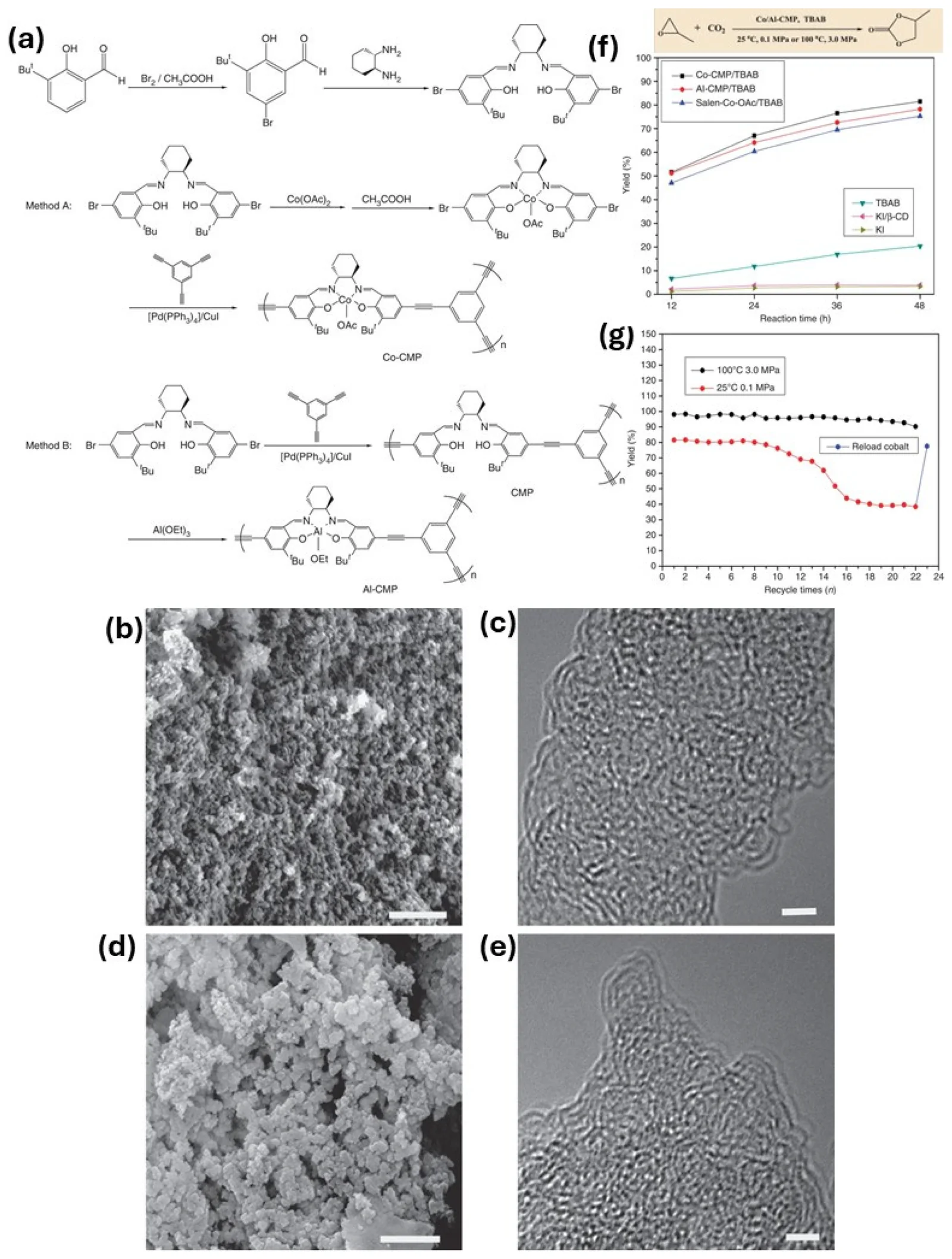
(**a**) Steps involved in the synthesis of the Co-CMP (Method A) and Al-CMP (Method B), (**b**,**d**) SEM images of the Co-CMP (**b**) and Al-CMP (**d**) (scale bar: 500 nm), and HR-TEM images of Co-CMP (**c**) and Al-CMP (**e**) (scale bar: 2 nm). (**f**) Catalytic testing of the Co-CMP and Al-CMPs for the conversion of PO to PC as a function of time under atmospheric pressure and room-temperature conditions, and (**g**) recycling stability of Co-CMP evaluated under different experimental conditions, adapted from [58]. Copyright 2013 Springer Nature Limited.

**Figure 3:**
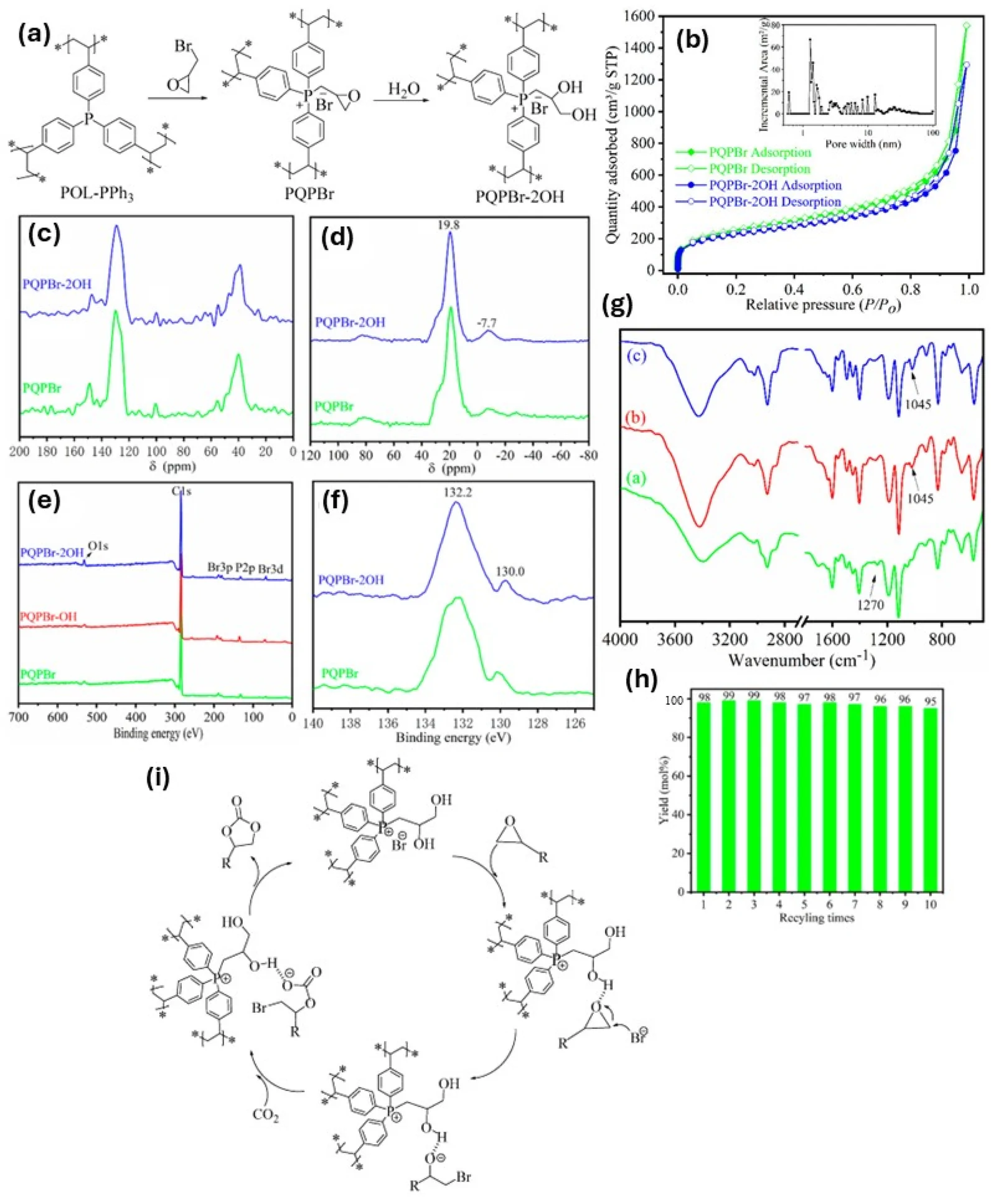
(**a**) Schematic of the synthesis of the PQPBr-2OH catalyst, (**b**) Nitrogen adsorption–desorption isotherms of PQPBr and PQPBr-2OH, and the pore size distribution of PQPBr-2OH (inset), (**c**) solid-state ^13^C-NMR spectra of PQPBr and PQPBr-2OH, (**d**) solid-state ^31^P-NMR spectra of PQPBr and PQPBr-2OH, (**e**) XPS survey spectra of PQPBr, PQPBr-OH, and PQPBr-2OH, (**f**) high-resolution P 2p XPS spectra of PQPBr and PQPBr-2OH, (**g**) FT-IR spectra of (**a**) PQPBr, (**b**) PQPBr-OH, and (**c**) PQPBr-2OH, (**h**) catalytic reusability and recycling performance of PQPBr-2OH, and (**i**) proposed reaction mechanism for the cycloaddition of CO_2_ with epoxides catalyzed by PQPBr-2OH, adapted from [91]. Copyright 2020 MDPI.

**Figure 4:**
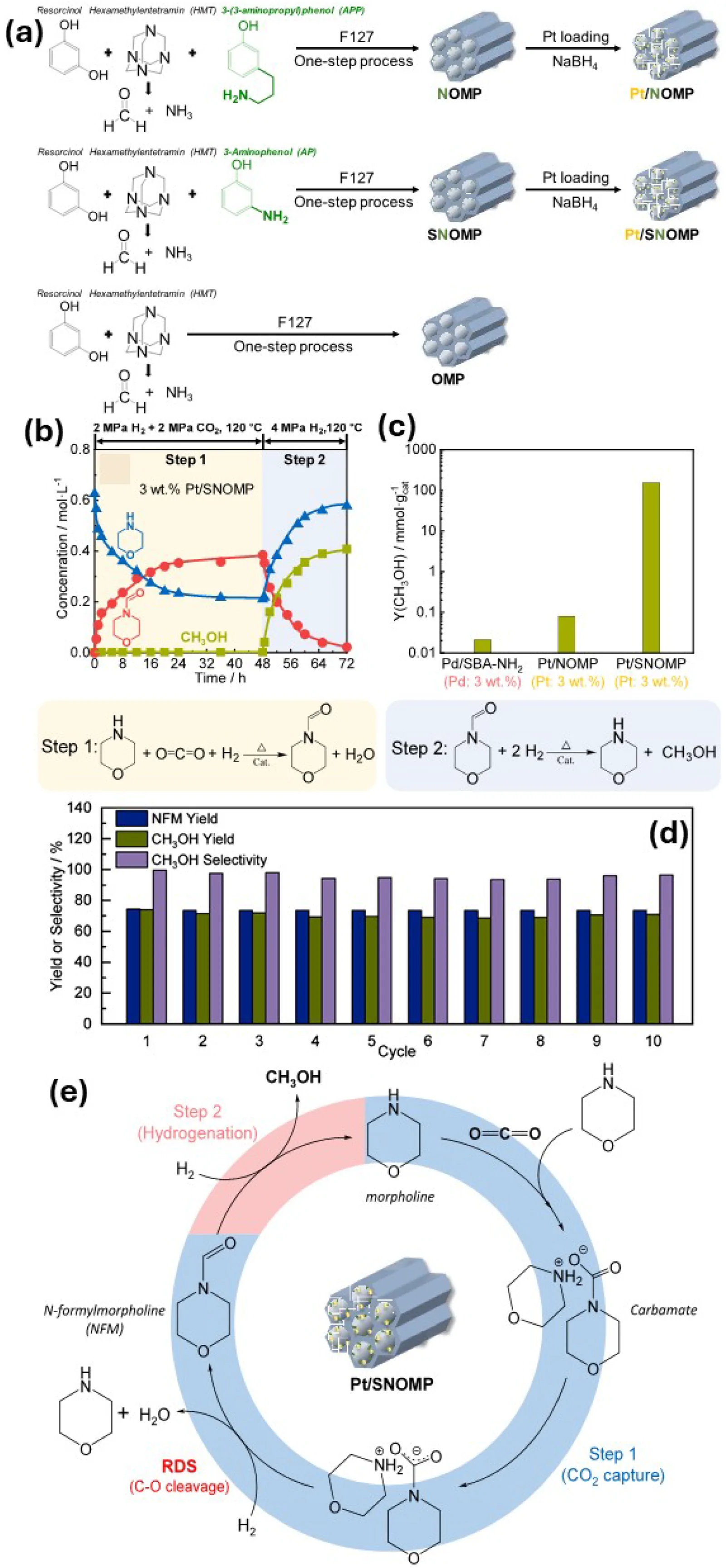
(**a**) Illustration for the synthesis of the bifunctional Pt/NOMP and Pt/SNOMP catalysts, as well as the parent OMP support, (**b**,**c**) catalytic activity of Pt/SNOMP in the morpholine-assisted CO_2_ hydrogenation to methanol, (**b**) reaction kinetics as a function of time of the one-pot, two-step reaction catalyzed by 3 wt% Pt/SNOMP, (**c**) comparison of methanol yields achieved with Pt/SNOMP in a liquid-phase system and conventional bifunctional catalysts (Pt/NOMP and Pd/SBA-NH_2_) in gas-phase fixed-bed reactor (note: the methanol yield axis is presented on a logarithmic scale), (**d**) recycling performance evaluated over ten consecutive reaction cycles of the 3 wt% Pt/SNOMP catalyst, and (**e**) proposed reaction mechanism for the morpholine-assisted CO_2_ hydrogenation to methanol over the Pt/SNOMP catalyst, adapted from [95]. Copyright 2026 ACS.

**Figure 5:**
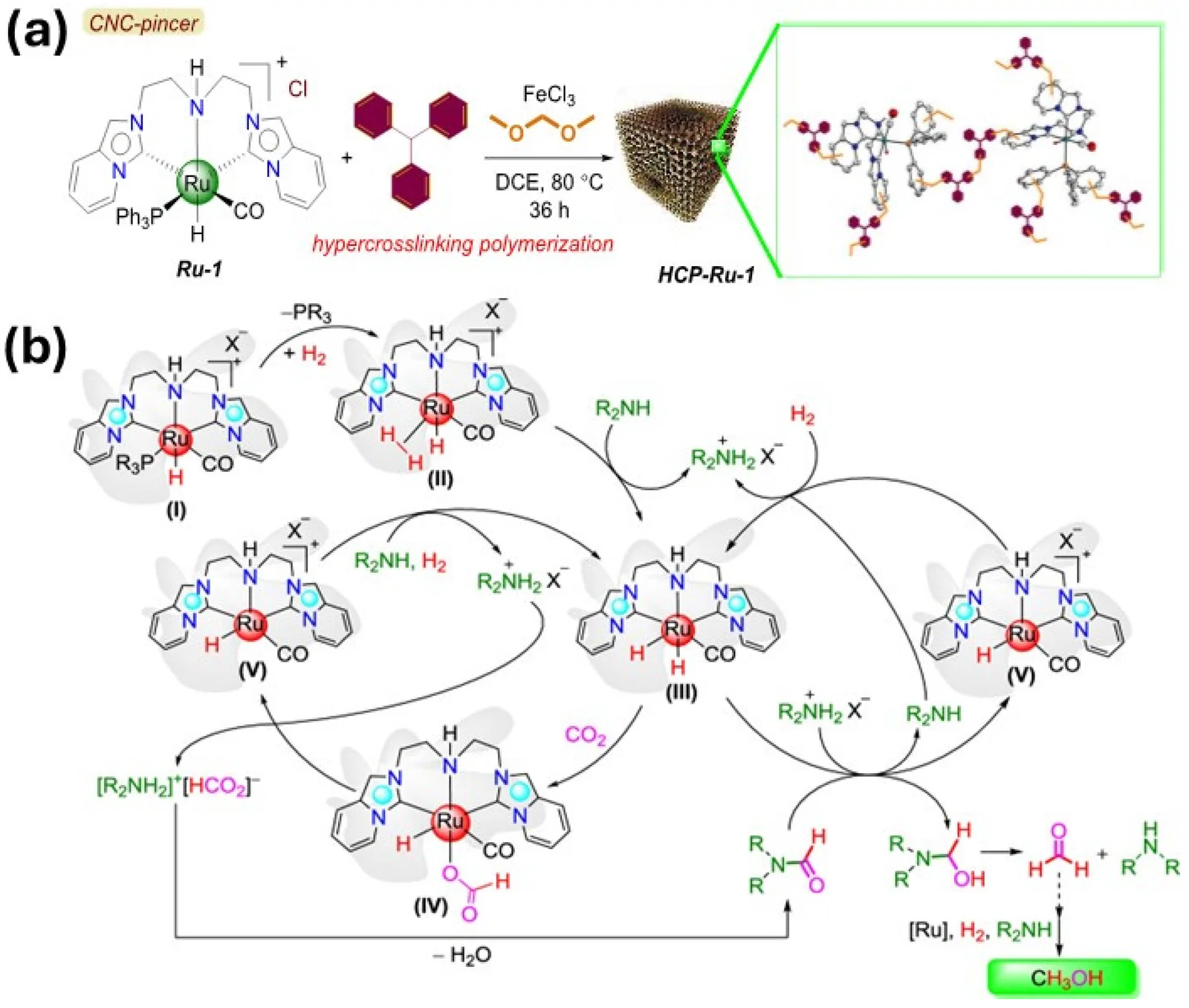
(**a**) Development of Ru-NHC-based homogeneous and heterogeneous catalysts for amine-assisted CO_2_ hydrogenation to methanol and (**b**) proposed catalytic cycle for CO_2_ hydrogenation to CH_3_OH using PEHA (represented as R_2_NH here) as an amine, reproduced with permission from [100]. Copyright 2024 ACS.

**Figure 6:**
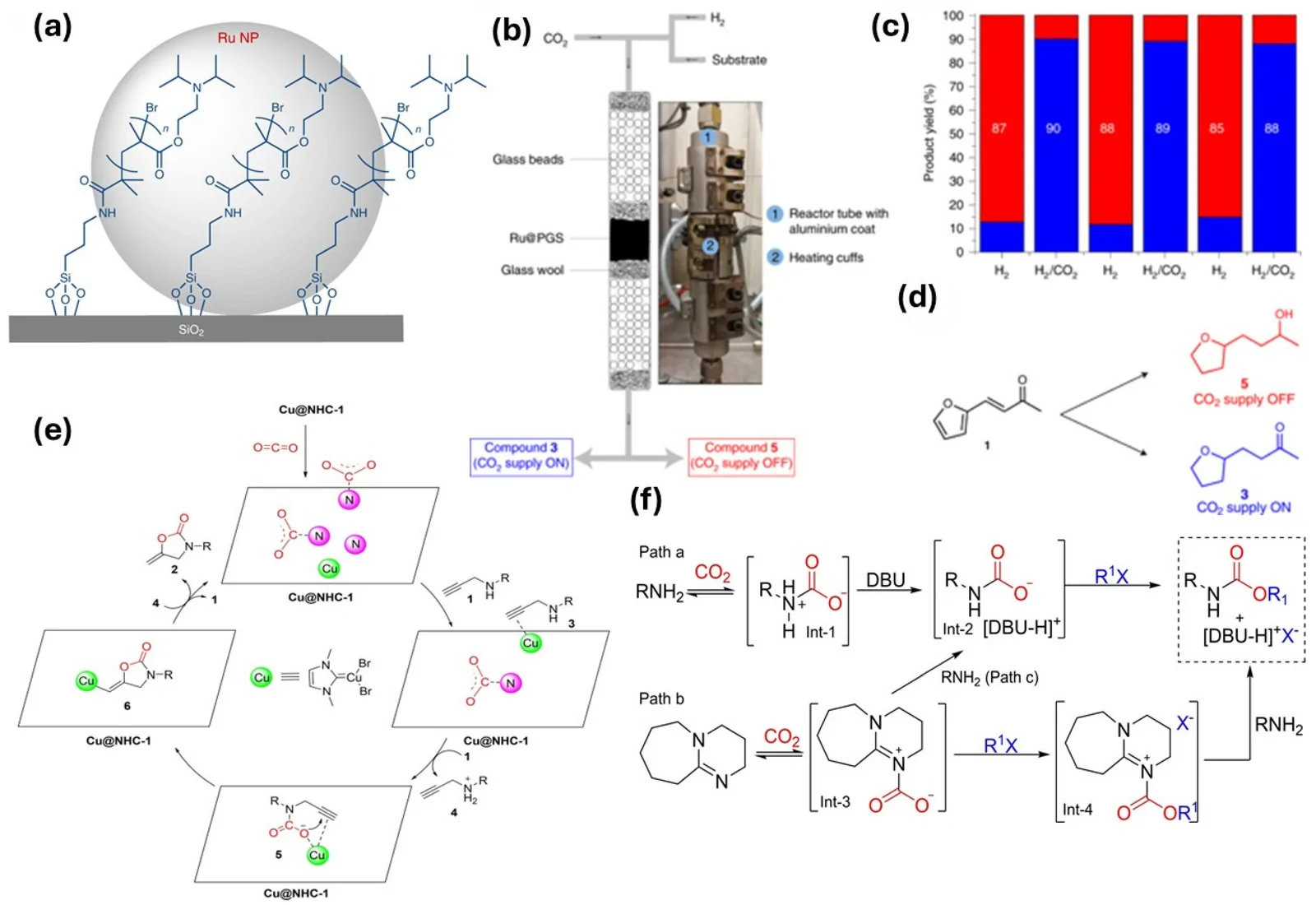
Different types of CO_2_ conversion reactions catalyzed by functional polymer-based materials, along with their proposed mechanisms and evaluation of switchable catalysis under continuous-flow conditions: (**a**) Schematic structure of the Ru@PGS catalyst featuring Ru nanoparticles supported on amine-functionalized PGS, (**b**) schematic illustration and photograph of the continuous-flow reactor setup, (**c**) hydrogenation of furfural acetone over Ru@PGS under H_2_ and H_2_/CO_2_ atmospheres, and (**d**) reversible selectivity switching controlled by the feed gas composition, enabling the selective production of saturated alcohols or ketones in high yields with conversions above 99%. Adapted from [110]. Copyright 2021 Nature. (**e**) Cu@NHC-1–catalyzed carboxylative cyclization of propargylamine using low-concentration CO_2_, reproduced with permission from [114]. Copyright 2023 Wiley-VCH GmbH. (**f**) Carbamate synthesis over PS-DBU, reproduced with permission from [109]. Copyright 2023 Wiley-VCH GmbH.

## Data Availability

No new data were created or analyzed in this study. Data sharing is not applicable.
